# Transcriptional regulatory network triggered by oxidative signals configures the early response mechanisms of japonica rice to chilling stress

**DOI:** 10.1186/1471-2229-10-16

**Published:** 2010-01-25

**Authors:** Kil-Young Yun, Myoung Ryoul Park, Bijayalaxmi Mohanty, Venura Herath, Fuyu Xu, Ramil Mauleon, Edward Wijaya, Vladimir B Bajic, Richard Bruskiewich, Benildo G de los Reyes

**Affiliations:** 1 School of Biology and Ecology, University of Maine, Orono, ME 04469, USA; 2 South African National Bioinformatics Institute, University of the Western Cape, Bellville 7535, South Africa; 3 Crop Research Informatics Laboratory, International Rice Research Institute, Los Banos, Laguna, Philippines; 4 Department of Chemical and Biomolecular Engineering, National University of Singapore, 117576, Singapore; 5 Computational Biology Research Center, AIST Tokyo Waterfront, 2-41-6 Aomi, Koto-ku, Tokyo 135-0064, Japan; 6 Computational Bioscience Research Center, King Abdullah University of Science and Technology, Thuwal, Kingdom of Saudi Arabia

## Abstract

**Background:**

The transcriptional regulatory network involved in low temperature response leading to acclimation has been established in *Arabidopsis*. In japonica rice, which can only withstand transient exposure to milder cold stress (10°C), an oxidative-mediated network has been proposed to play a key role in configuring early responses and short-term defenses. The components, hierarchical organization and physiological consequences of this network were further dissected by a systems-level approach.

**Results:**

Regulatory clusters responding directly to oxidative signals were prominent during the initial 6 to 12 hours at 10°C. Early events mirrored a typical oxidative response based on striking similarities of the transcriptome to disease, elicitor and wounding induced processes. Targets of oxidative-mediated mechanisms are likely regulated by several classes of bZIP factors acting on as1/ocs/TGA-like element enriched clusters, ERF factors acting on GCC-box/JAre-like element enriched clusters and R2R3-MYB factors acting on MYB2-like element enriched clusters.

Temporal induction of several H_2_O_2_-induced bZIP, ERF and MYB genes coincided with the transient H_2_O_2 _spikes within the initial 6 to 12 hours. Oxidative-independent responses involve *DREB/CBF*, *RAP2 *and *RAV1 *factors acting on DRE/CRT/rav1-like enriched clusters and bZIP factors acting on ABRE-like enriched clusters. Oxidative-mediated clusters were activated earlier than ABA-mediated clusters.

**Conclusion:**

Genome-wide, physiological and whole-plant level analyses established a holistic view of chilling stress response mechanism of japonica rice. Early response regulatory network triggered by oxidative signals is critical for prolonged survival under sub-optimal temperature. Integration of stress and developmental responses leads to modulated growth and vigor maintenance contributing to a delay of plastic injuries.

## Background

Plants in temperate environments are able to withstand sub-freezing conditions by cold acclimation, whereas those that are adapted to tropical or sub-tropical environments can only maximally endure milder chilling temperatures [1]. *Arabidopsis *has been a particularly well scrutinized model system for low temperature stress response mechanisms because of its ability to cold acclimate [2-4]. Cold acclimation is a manifestation of the coordinated function of a large number of genes under the control of few transcriptional regulators that respond directly to low temperature signals through abscisic acid (ABA) independent signal transduction [5,6].

The established resources for functional genomics in *Arabidopsis *facilitated a systematic approach for assembling the components of regulatory networks involved in cold acclimation as well as those involved in similar types of abiotic stresses [7]. The most well documented mechanism involves a class of ERF transcription factors (TF) known as the *DREB/CBF*, which interact with the DRE/CRT cis-elements in the promoters of their downstream target genes to execute a highly coordinated transcriptional response to low temperature signals [8-10]. The *DREB/CBF *regulon is a large cluster consisting of more than a hundred genes with associated sub-regulons controlled by *RAP2.1 *and *RAP2.6 *[8,9,11]. Acting upstream is the MYC-type *DREB/CBF *activator *ICE1*, which is regulated by phosphorylation [12]. Discovery of other regulons that function during cold acclimation is a continuing story. Recently, a *DREB/CBF*-independent regulon controlled by a zinc finger TF (*ZAT12*) was discovered [3,8]. *ZAT12 *acts in parallel to the *CBF/DREB *pathway although each mechanism appears to activate overlapping sets of *COR *genes. Additional regulatory clusters controlled by *RAV1 *TF have also been inferred based on transcriptome profiles of overexpression lines [3,9].

Rice is another important model for dissecting stress response regulatory networks because of its 'finished' genome sequence and rapidly developing functional genomics resources. Unlike the freeze-tolerant *Arabidopsis*, most rice cultivars are irreversibly injured by prolonged exposure to temperatures above 10°C (chilling), particularly at early seedling stage. However, many japonica cultivars are moderately adapted to temperate climates and they generally exhibit lesser degrees of chilling sensitivity than most tropical indica cultivars. Previous efforts to profile the low temperature transcriptome of japonica rice was based on the responses to 4°C [13], which is below the typical seedling stage LT_50 _of 13°C for even the most chilling tolerant japonica cultivars [14-16]. The scope of transcriptional changes that occur when japonica rice is exposed to chilling (10°C) has been investigated only recently in an effort to establish a more agronomically meaningful picture of response mechanisms [14,17,18]. In our earlier analysis of about 6,000 genes, we recognized peculiar trends suggesting that transient oxidative stress during the initial 24 hours triggered an '*early response*' regulatory network that seems to play an important role in short-term adaptive responses [17]. To dissect the various components of this network and other aspects that are independent of oxidative signals, we performed two parallel genome-wide expression profiling experiments comparing the responses of japonica rice to chilling (10°C) and cold-independent mimic of oxidative stress.

With the systems level approach employed in this study, we established a broader picture of the hierarchical organization and compositional complexity of upregulated transcriptional network supported by direct physiological evidence that oxidative signal caused by transient increases in intracellular H_2_O_2 _is a primary trigger for network activation. Potential agronomic significance of oxidative-mediated network was established by integrating different layers of information derived from genome-wide, physiological and whole-plant level analyses. The results of this study indicate that expression of early response regulatory network is a critical determinant of the ability of japonica rice for prolonged survival under sub-optimal temperature conditions. This is achieved by integrating defense and growth related responses leading to the ability to delay the progression of irreversible stress injuries by maintenance or enhancement of vigor.

## Results and Discussion

### Profile of chilling stress response transcriptome of japonica rice

Two parallel transcript profiling experiments were performed using the NSF-45K oligonucleotide microarray [19]. In the first experiment (GSE8767), gene expression in cv. Nipponbare was monitored during a 96-hour duration at 10°C covering both early (0.5, 2, 4, 6, 12, 18, 24 hours) and late (36, 48, 96 hours) responses. In the second experiment (GSE10062), gene expression in response to applied H_2_O_2 _(4 mM) after 1, 3 and 6 hours of treatment at optimum temperature (28°C) was investigated also in cv. Nipponbare. The overall assumption was that gene activation by oxidative signals and those triggered by other physiological perturbations can be inferred by subtracting the composition of one dataset from the other.

**Figure 1:**
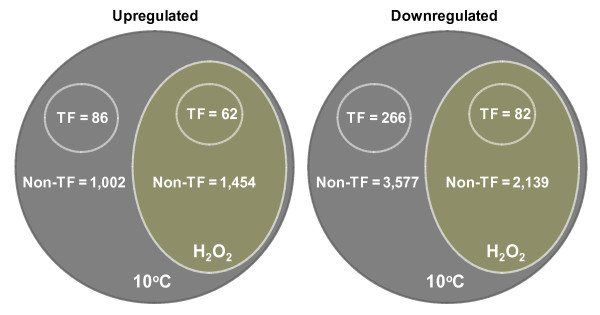
**General features of the chilling stress response transcriptome of japonica rice (cv. Nipponbare) based on the analysis of expression profiles across 10 time points**. The composition of the non-overlapping upregulated (2,604 genes) and downregulated (6,064 genes) components are indicated. Venn diagram also shows the overlap between chilling and H_2_O_2 _response transcriptomes. For the upregulated component, 1,088 genes (86 transcription factors + 1,002 non-transcription factors) were induced by chilling but not by exogenous H_2_O_2 _and 1,516 genes (62 transcription factors + 1,454 non-transcription factors) were induced by both chilling and exogenous H_2_O_2_. For the downregulated component, 3,843 genes (266 transcription factors + 3,577 non-transcription factors) were repressed by chilling but not by exogenous H_2_O_2 _and 1,516 genes (82 transcription factors + 2,139 non-transcription factors) were repressed by both chilling and exogenous H_2_O_2_.

Chilling stress has massive effects on the transcriptome, which is comprised of 8,668 genes that exhibited temporal changes during a 96-hour duration at 10°C. The upregulated components were identified by querying the whole microarray dataset at an arbitrary threshold of log_2 _≥ 1.8 (p < 0.05), revealing a total of 2,604 unique genes that were induced within a duration of at least two consecutive time points. Nearly 60% (1,516) of these genes were also induced by 4 mM H_2_O_2 _(p < 0.05) in at least one time point (Figure 1). This overlap was assumed to represent the components of chilling stress response transcriptome that were activated by an oxidative-mediated regulatory mechanism.

The downregulated dataset does not overlap with the upregulated dataset. It includes all the genes (total = 6,064) with expression values of log_2 _≤ -1.8 (p < 0.05) in at least one time point and without any value equal to or greater than the upregulated threshold (log_2 _1.8) in any one time point. Of these, 25% (1,516) were also downregulated by 4 mM H_2_O_2 _(Figure 1). These genes were assumed to represent the components of chilling stress transcriptome that were negatively regulated by an oxidative-mediated mechanism. The composition and temporal profiles of the downregulated group were quite complex. A summary of the distribution of these genes according to broad functional categories is presented (see Additional file 1).

Downregulation appears to have major impact on cellular processes related to growth, development and morphogenesis, cell division, metabolism and transport mechanisms. Due to the complexity of the temporal patterns exhibited by this group of genes, the assembly of underlying regulatory network and their linkages with the upregulated network will be described in a separate report based on the analysis of transcription factor overexpression lines.

### Major regulators of chilling stress response transcriptome

**Figure 2:**
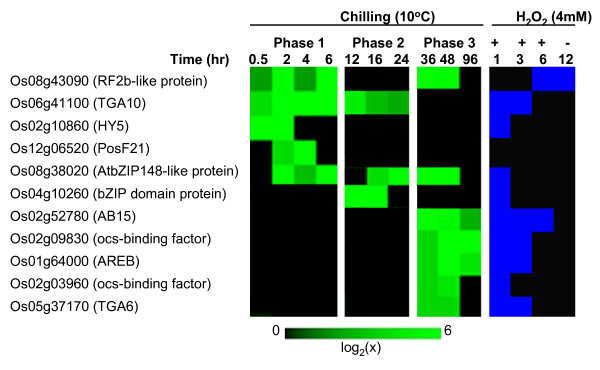
**Expression matrix of chilling upregulated bZIP transcription factors**. Expression values are represented by the means of two independent biological replicates (p < 0.05). Members of this group include the genes that were upregulated (≥log_2 _[1.8]) in at least two consecutive time points and not downregulated in any time point. Expression profiles in response to exogenous H_2_O_2 _are presented in a binary format, where the upregulated and not upregulated time points were assigned a value of 1 (blue) and 0 (black), respectively. Locus IDs are according to TIGR rice genome annotation.

**Figure 3:**
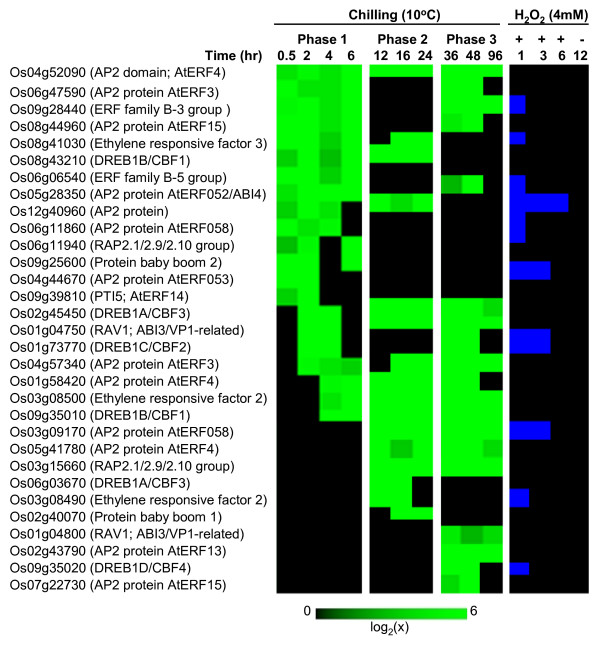
**Expression matrix of chilling upregulated ERF transcription factors**. Expression values are represented by the means of two independent biological replicates (p < 0.05). Members of this group include the genes that were upregulated (≥ log_2 _[1.8]) in at least two consecutive time points and not downregulated in any time point. Expression profiles in response to exogenous H_2_O_2 _are presented in a binary format, where the upregulated and not upregulated time points were assigned a value of 1 (blue) and 0 (black), respectively. Locus IDs are based on TIGR rice genome annotation.

**Figure 4:**
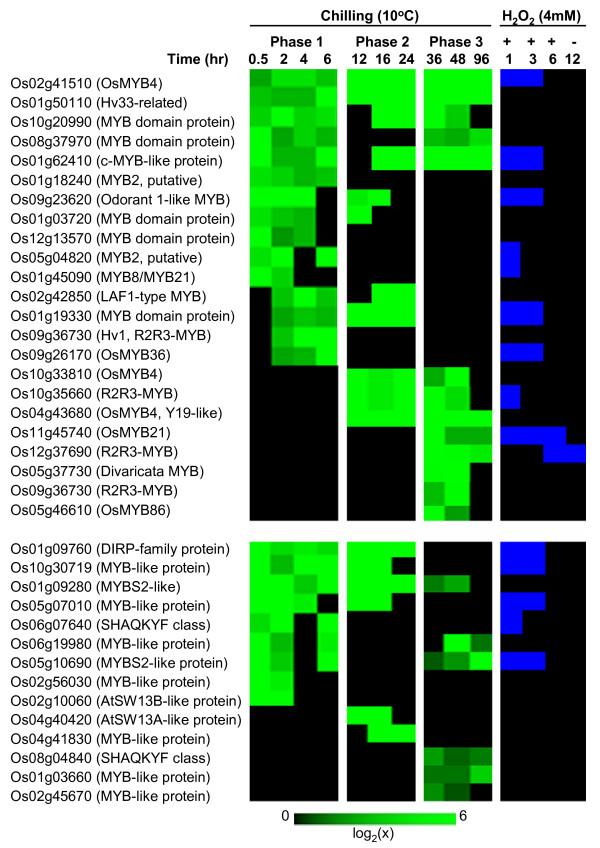
**Expression matrix of chilling upregulated MYB transcription factors**. Expression values are represented by the means of two independent biological replicates (p < 0.05). Members of this group include the genes that were upregulated (≥log_2 _[1.8]) in at least two consecutive time points and not downregulated in any time point. Expression profiles in response to exogenous H_2_O_2 _are presented in a binary format, where the upregulated and not upregulated time points were assigned a value of 1 (blue) and 0 (black), respectively. Locus IDs are based on TIGR rice genome annotation. Top panel = R2R3 group; Bottom panel = R1 (MYB-like) group.

Transcription factors (TF) link stress signals to the downstream genes that execute cellular defense processes [20]. Of the 2,604 chilling upregulated genes, about 6% (148) are TFs (Figure 1, Table 1), which were identified based on the classification of the Database of Rice Transcription Factors [21,22]. These genes represent more than 7% of the total TFs encoded by the rice genome and include members of AP2/ERF, bZIP, MYB, WRKY, bHLH and NAC families [3,6,23-26]. TFs exhibited complex expression patterns characterized by waves of induction initiating at different time periods (Figures 2, 3, 4; see Additional files 2, 3, 4). For instance, activation of the '*early rapid response*' group (phase-1) occurred during the initial 6 hours, while those that belong to the '*early slow response*' group (phase-2) occurred between 6 and 24 hours. Few TFs exhibited '*late response*' profiles (phase-3) with no significant induction until after 24 hours. Majority of chilling upregulated TFs were activated at phase-1 and phase-2, indicating that the critical transcriptional changes occurred during the initial 24 hours.

**Table 1:** Classification of chilling upregulated transcription factors by family.

TF Family	Number of genes induced only by chilling	Number of genes induced by both chilling and H_2_O_2_	% of total genes within family
bZIP	1	10	10.1

WRKY	10	7	16.3

NAC	16	7	14.6

MYB	23	14	17.7

bHLH	17	12	18.5

AP2/ERF	20	11	17.8

*Per cent chilling induced genes relative to genome-wide total for each family based on the most recent annotation of the Nipponbare genome by Gramene and RAP-DB.

Total per family: 109 (bZIP), 111 (WRKY), 157 (NAC), 209 (MYB), 157 (bHLH), 174 (AP2/ERF).

Induction waves were either short-lived or long-lived events lasting between 2 to 60 hours. Some genes are activated multiple times based on interrupted patterns (Figures 2, 3, 4). Multi-phasic profiles reflect the temporal hierarchy of regulatory networks operating at 10°C and the complexity of signals that trigger the activity of regulatory clusters. Apparently, phase-1 TFs are closest to the original signal and are likely to function as primary switches that trigger subsequent cascade of gene activation. Reactivation of some phase-1 TFs at later time points suggests that multiple signals are acting on the same TF. Phase-2 and phase-3 TFs are likely controlled by other phase-1 TFs that respond directly to the primary signal. Similar hierarchical organization of stress related TFs has been shown earlier. For example, *DREB/CBF *controls two other types of ERF genes (*RAP2.1, RAP2.6*) involved in the activation of DRE/CRT element-containing genes as well as an R2R3-type MYB that control other potential sub-regulons related to *CBF/DREB *network [3,9,27].

### bZIP family

Members of the bZIP family of TFs have important roles in ABA response and regulation of oxidative and pathogen defense responses [5,28]. A total of 11 genes from this family were upregulated by chilling (Figure 2), representing five sub-classes based on recent phylogenetic classification [24,29]. The '*early response*' (phase-1 and phase-2) genes belong to Group-D associated with oxidative stress and defenses against pathogens (Os06g41100: *TGA10*), Group-H associated with photomorphogenesis (Os02g10860: *HY5*, Os12g06520: *PosF21*), Group-I associated with GA response (Os08g43090: *RF2b*-group, Os04g10260: bZIP domain protein), and Group-S associated with stress response and sucrose signaling (Os08g38020: *AtbZIP148*-like) and a novel gene that appears to be specific to rice (Os0410260). The ABA-associated bZIP genes belonging to Group-A (Os02g52780: *ABI5*, Os01g64000: *AREB*) were not induced until after 24 hours (phase-3). Several Group-D (Os05g37170: *TGA6*) and Group-S (Os02g09830, Os02g03960: ocs-binding proteins) genes were also induced at phase-3. Majority of chilling induced bZIP genes were also induced by H_2_O_2 _(Figure 2). Many (Os06g41100, Os05g37170, Os02g09830, Os02g03960) are known to interact with as1/ocs/TGA-like elements involved in H_2_O_2_, auxin, disease, salicylic acid and jasmonic acid regulated gene expression [30-33].

**Figure 5:**
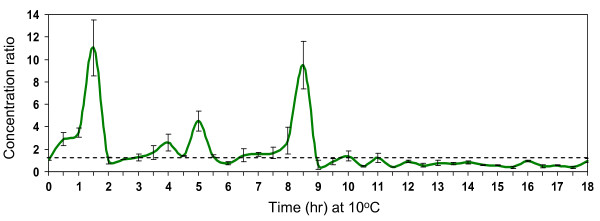
**Profile of chilling-induced H_2_O_2 _accumulation in leaves of Nipponbare seedlings at the V_3 _stage**. H_2_O_2 _level was measured at 30 minutes interval in chilling stressed (10°C) and control (28°C) seedlings. Values represent the ratio of the concentrations in chilling stressed and control plants at each time point ([nmole chilling]/[nmole control]). Peak values at 1.5, 4, 5 and 8.5 hours were significantly greater than 1 at p < 0.01, n = 3 (SE shown as error bars).

ABA induces H_2_O_2 _synthesis in guard cells via NADPH oxidase [34,35]. This was indicated by impaired stomatal closure in double mutants for NADPH oxidase *AtrbohD *and *AtrbohF *genes, which was reversed by exogenous H_2_O_2 _[36]. ABA-related bZIP genes of rice (Os02g52780, Os01g64000) were also induced by H_2_O_2 _but their late induction implies direct consequences of ABA rather than oxidative signaling. We observed that rice seedlings had visible symptoms of leaf rolling not earlier than 24 hours after exposure to 10°C (data not shown), which was coincident with the temporal expression of ABA-related bZIP genes at phase-3. This pattern indicates that oxidative-mediated gene expression occurred much earlier (within the initial 6 hours) than those mediated by ABA (after 24 hours), and that ROS (H_2_O_2_) was the primary signal that integrates early responses (Figure 5). This is consistent with our earlier observations that many chilling induced genes that were also induced by ABA were '*late response*' genes [[17], unpublished data].

### AP2/ERF family

About 18% (31) of AP2/ERF genes of rice were induced by chilling (Table 1; Figure 3). Most are ERF-type belonging to nine phylogenetic groups [3,5,20,37]. Genes involved in indole alkaloid metabolism (ORCA-type) and responses to diseases, wounding and elicitors such as ethylene, salicylic acid and jasmonic acid comprise one of the larger classes which include Group-VI/subfamily B-5 (Os06g06540), Group-VIII/subfamily B-1 (Os04g52090, Os06g47590, Os05g41780, Os06g47590, Os01g58420, Os04g57340), and Group-IX/subfamily B-3 (Os02g43790, Os07g22730, Os08g44960, Os09g39810). Many are known to interact with GCC-box and jasmonic acid response element (JAre) [38-41]. Genes in this category were expressed in all three phases but only few were responsive to H_2_O_2 _(Figure 3).

Another large category of chilling-induced ERF represent a class of genes homologous to the low temperature, dehydration and salinity stress regulators in *Arabidopsis *belonging to Group-II/subfamily A-5 (*RAP2.1/2.9/2.10 *group: Os03g15660, Os06g11940), Group-III/subfamily A-5 (*DREB/CBF *group: Os08g43210, Os02g45450, Os01g73770, Os09g35010, Os06g03670, Os09g35020) and Group-VII/subfamily B-2 (Os03g08500, Os03g08490, Os12g40960). Members of these groups interact with DRE/CRT and related cis-elements [6,42,43]. Only few of these genes were induced by H_2_O_2_, suggesting that this class of ERF is not a direct target of oxidative signaling although cross-talk is possible as indicated by few *DREB/CBF *genes that responded to the H_2_O_2 _treatment (Figure 3). Specific *DREB/CBF *genes from Group-III were activated at phase-1 (Os02g45450: *DREB1A/CBF3*, Os08g43210/Os09g35010: *DREB1B/CBF1*, Os01g73770: *DREB1C/CBF2*), phase-2 (Os06g03670: *DREB1A/CBF3*) and phase-3 (Os09g35020: *DREB1D/CBF4*), indicating that different members respond to specific signals at various stages of stress.

The third category of chilling induced ERF genes are associated with growth regulation and ABA signaling from Group-I/subfamily A-6 (Os04g44670, Os06g11860, Os03g09170), Group-IV/subfamily A-2 (Os05g28350) and Group-X/subfamily B-4 (Os09g28440) [44,45]. Genes in this category were activated either at phase-1 or phase-2 and many were also induced by H_2_O_2 _(Figure 3). Early induction appears to be a consequence of oxidative signaling rather than ABA, given that ABA response does not seem to occur until after 24 hours after chilling exposure.

Several non-ERF-type TFs were also induced by chilling, including the RAV1-type *ABI3/VP1 *homologs (Os01g04750, Os01g04800), genes encoding baby boom-like proteins (Os09g25600, Os02g40070) and a novel *ERF3*-like gene (Os08g41030) (Figure 3). *RAV1*-type TFs function in low temperature response and circadian regulation [5,9], while the baby boom-like proteins are involved in development [20]. Rice seedlings at three-leaf (V_3_) stage, which is about 12 days after germination, were used in the microarray experiments. Activation of *RAV1*-type and baby boom-type transcription factors reflects the cross-talk that typically occurs between low temperature response and developmental processes and this is probably important to integrate signals necessary for normal seedling growth and immediate defenses to stress.

Homologs of ERF genes that function maximally during cold acclimation at 4°C in *Arabidopsis *were activated at milder (10°C) temperature [27]. The regulatory clusters and physiological consequences are likely to be different under non-acclimating (10°C) and acclimating (4°C) temperature regimes based on the observation that *CBF/DREB *induction at 10°C tend to be relatively short-lived and less robust [11]. Nevertheless, the transcriptional network at 10°C provides an agronomically meaningful view of response mechanisms since the LT_50 _for most japonica cultivars is about 13°C [14,16,46]. ERFs involved in disease response via oxidative, jasmonic acid and salicylic acid signaling are also involved in chilling stress response. However, they appear to act on a different set of target genes from those regulated by *DREB/CBF*.

### MYB family

MYB domain proteins represent the largest class of TFs involved in cell cycle regulation, cell fate determination and responses to environmental stresses [47-49]. MYB is also the largest group of chilling induced TFs with 37 members (Figure 4) belonging to three sub-families [50]. The two largest are the R2R3 subfamily (Os02g41510, Os01g50110, Os01g18240, Os09g23620, Os05g04820, Os01g45090, Os09g36730, Os09g26170, Os10g33810, Os10g35660, Os04g43680, Os11g45740, Os12g37690, Os09g36730, Os05g46610) and R1-MYB/MYB-like subfamily (Os01g09760, Os10g30719, Os01g09280, Os05g07010, Os06g07640, Os06g19980, Os05g10690, Os02g56030, Os02g10060, Os04g40420, Os04g41830, Os08g04840, Os01g03660, Os02g45670). Many R2R3 genes are known to function in cold, salinity and ABA response mechanisms [51-53]. A total of 24 MYB genes were activated at phase-1, 5 at phase-2 and 8 at phase-3 (Figure 4). Phase-1 MYB genes tend to be more responsive to exogenous H_2_O_2 _than those in phase-2 and phase-3, indicating that '*early response*' genes are possible direct targets of oxidative signals including the *OsMYB4 *(Os02g41510) that we previously reported [17].

### WRKY family

WRKY TFs function as positive or negative regulators of defenses against pathogens and herbivores via salicylic acid and jasmonic acid signaling pathways [54-58]. Their precise role in abiotic stress response regulatory network is not fully understood [59,60]. A total of 17 members of this family were induced by chilling (see Additional file 2). Most are '*early response*' genes, 10 of which were induced at phase-1 and 5 at phase-2. Phase-1 genes include several known regulators of salicylic acid and jasmonic acid mediated disease response (Os01g14440: *OsWRKY1v2*, Os09g24070: *OsWRKY62*, Os03g55164: *OsWRKY4*, Os05g39720: *OsWRKY53*, Os11g45850: *OsWRKY40*, Os05g04640: *OsWRKY5*), and few have recently been shown to function in ABA-mediated gene expression (Os01g61080: *OsWRK24*, Os11g29870: *OsWRKY72*). *OsWRKY24 *is particularly interesting because it was recently shown to act as repressor of ABA-mediated induction of *HVA22 *[60,61]. Upregulation of this gene at phase-1 is quite consistent with our current data showing that ABA-regulated expression of *ABI5 *and *AREB *genes occurred largely at phase-3. *OsWRKY24 *appears to act as repressor of ABA-inducible genes during the early stages of chilling stress and it is likely involved in the integration of responses to oxidative and ABA signals.

Phase-2 WRKY genes are mostly disease-related (Os05g27730; *OsWRKY53*; Os05g50610: *OsWRKY8v2*, Os01g51690: *OsWRKY26*) and herbivory-related (Os03g58420: *OsWRKY6*). Downregulation of *OsWRKY53 *by ABA was reported recently [61]. *OsWRKY53 *expression lasted only up to phase-2 which is probably due to increased ABA levels at phase-3. Three of the phase-2 WRKY genes were induced by H_2_O_2 _and they are probably secondary targets of oxidative signaling through other TFs that are induced at phase-1. Their activation by H_2_O_2 _may also be mediated by salicylic acid or jasmonic acid [62]. It is possible that some of the phase-1 and phase-2 WRKY genes are involved in repression of gene expression to fine-tune the interaction of early (oxidative) and late (ABA) signaling mechanisms. Enrichment of W-box-like motifs among downregulated genes are consistent with this hypothesis (data not shown).

### bHLH family

TFs from the bHLH family are involved in plant development, morphogenesis, circadian regulation and stress response [63]. MYC-type factors are involved in ABA-regulated gene expression [12,26,37]. About 19% (29) of rice bHLH genes were induced by chilling, 18 of which were at phase-1, 5 at phase-2 and 6 at phase-3. Half of '*early response*' bHLH genes were responsive to exogenous H_2_O_2 _and their profiles were characterized by multiple waves reflecting possible roles in combinatorial control mechanisms (see Additional file 3).

### NAC family

NAC (NAM, ATAF1/2, CUC2) genes encode a group of plant-specific TFs involved in growth and hormone signaling, apical meristem and floral development, senescence, disease response via salicylic acid and jasmonic acid and abiotic stress responses via ABA [23,64,65]. A total of 23 NAC genes were induced by chilling, including 21 '*early response*' (14 at phase-1 and 7 at phase-2) and 2 '*late response*' (Table 1; see Additional file 4). Only few of the NAC genes (mostly phase-1 genes) were also induced by H_2_O_2_.

### Stress induced H_2_O_2_: Temporal coincidence with transcription factor activation

Cellular concentrations of ROS including H_2_O_2 _are tightly regulated by balancing synthesis and degradation. Exposure to sub-optimal conditions often disturbs such balance [66,67]. At a critical level within specific cellular microdomains, H_2_O_2 _acts as secondary messenger that integrates a variety of responses associated with biotic and abiotic signals and growth and development [68-71]. We have shown in our previous report that exogenous H_2_O_2 _mimics to a certain degree the effect of chilling on gene expression by activating many chilling-associated ROS scavengers and other stress related genes when plants were treated with 4 mM H_2_O_2 _at ambient temperature [17]. We hypothesized that a large component of chilling stress transcriptome is a consequence of ROS (H_2_O_2_) acting as primary signals for the activation of '*early response*' regulatory networks. To address this hypothesis further, we profiled the temporal fluctuation of intracellular H_2_O_2 _in Nipponbare at 10°C and 28°C (Figure 5).

Based on the changes in [chilling]/[control] ratios across time, H_2_O_2 _levels were particularly high during the initial 12 hours. H_2_O_2 _profile was characterized by transient waves indicating the interplay of stress-induced synthesis and degradation. H_2_O_2 _spikes differ in peak heights and occurred in three phases, first during the initial 2 hours followed by two more spikes between 4 and 6 hours and between 8 and 9 hours. After about 12 hours relative concentrations in stressed and control plants were stabilized as a consequence of the activation of ROS scavenging systems. Stabilization of H_2_O_2 _spikes was coincident with high expression of many ROS scavenging genes after 12 hours (see Additional file 4).

Of the 148 chilling upregulated TFs, 62 were also upregulated by exogenous H_2_O_2 _(Table 1). Activities at phase-1 and phase-2 mirror the temporal profile of cellular H_2_O_2 _accumulation. For instance, activation of many '*early response*' TFs that were also responsive to H_2_O_2 _coincided with the occurrence of H_2_O_2 _spikes within the initial 12 hours. These temporal coincidences provide an indirect but strong support that H_2_O_2 _is involved as a primary signal for the activation of '*early response*' regulatory network. H_2_O_2 _and other ROS are known to cause conformational changes of the DNA-binding domain of certain TFs [72-74]. We previously proposed that a constitutively expressed but inactive TF (e.g., MYB) could be activated by changes in redox status at 10°C and this possibly leads to rapid induction of its target TFs (e.g., *TGA10*) occurring at phase-1 [17]. Our current data support this hypothesis and provide a basis for possible concentration dependence of H_2_O_2_-inducible gene expression [73].

### Regulatory clusters associated with chilling upregulated transcription factors

**Figure 6:**
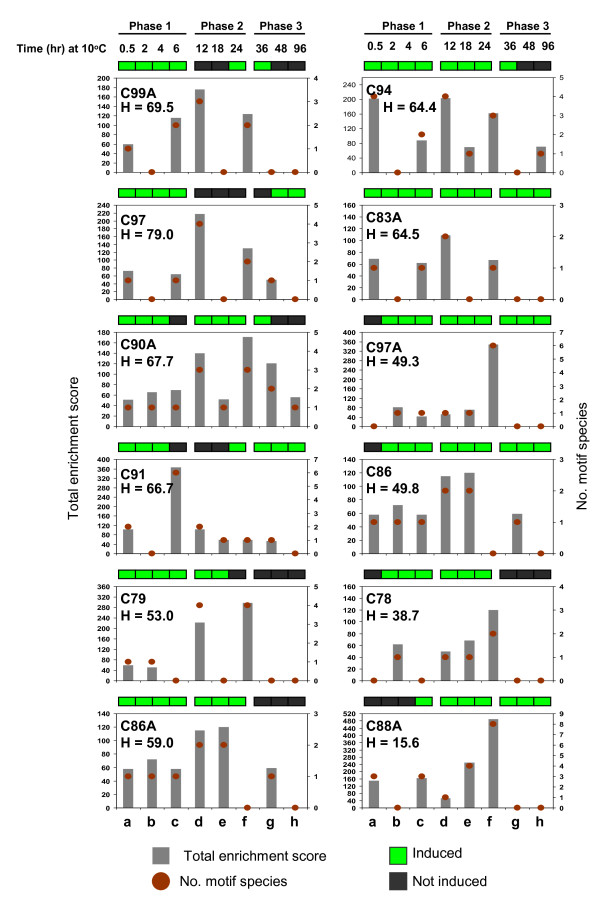
**Major classes of promoter cis-elements among the co-expressed gene clusters at phase-1**. Total enrichment score represents the sum of the % occurrences of all motif species that belong to the same broad category based on significant matches with known elements in promoter databases. Only the motif classes with occurrence of ≥50% in a given cluster were included in this analysis. The number of motif species for each class is also indicated for all clusters. The % of genes that were also induced by exogenous H_2_O_2 _(**H**) is given for each cluster. Consensus chilling-induced expression profiles based on the results of K-mean clustering are shown by the binary heat map on top of each graph. a = GCC-box/JAre-like (ERF), b = DRE/DRT/rav1-like (ERF), c = ABRE-like (bZIP), d = as1/ocs/TGA-like (bZIP), e = Myb2 box-like (R2R3-MYB), f = GARE/pyrimidine box-like (R1-MYB), g = W-box-like (WRKY), h = Myc2 box-like (bHLH).

**Figure 7:**
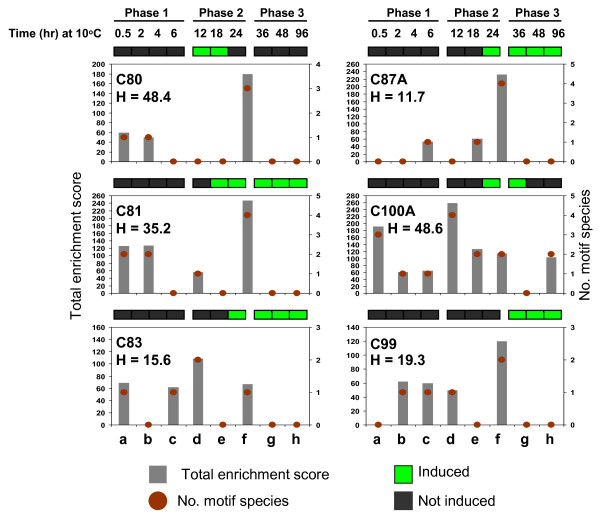
**Major classes of promoter cis-elements among the co-expressed gene clusters at phase-2 and phase-3**. Total enrichment score represents the sum of the % occurrences of all motif species that belong to the same broad category based on significant matches with known elements in promoter databases. Only the motif classes with occurrence of ≥50% in a given cluster were included in this analysis. The number of motif species for each class is also indicated for all clusters. The % of genes that were also induced by exogenous H_2_O_2 _(**H**) is given for each cluster. Consensus chilling-induced expression profiles based on the results of K-mean clustering are shown by the binary heat map on top of each graph. a = GCC-box/JAre-like (ERF), b = DRE/DRT/rav1-like (ERF), c = ABRE-like (bZIP), d = as1/ocs/TGA-like (bZIP), e = Myb2 box-like (R2R3-MYB), f = GARE/pyrimidine box-like (R1-MYB), g = W-box-like (WRKY), h = Myc2 box-like (bHLH).

Genes encoding non-transcription factor (NTF) proteins (2,456) were further classified based on expression profiles to establish the composition of various co-expressed groups (regulatory clusters) controlled by the different classes of TFs listed in Table 1. Genes were assigned to smaller groups by K-mean clustering to determine the closest similarities in expression [7]. A total of 18 clusters were established with members tightly distributed around the group mean (Figures 6, 7; see Additional file 5). Based on consensus expression profiles, genes can be assigned to a three-phase induction pattern similar to that observed among the various TF classes. Clusters C78, C79, C83A, C86, C86A, C88A, C90A, C91, C94, C97, C97A and C99A comprised the '*early rapid response*' group showing activation at phase-1 (Figure 6). Clusters C80, C81, C83, C87A and C100A comprised the '*early slow response*' group showing activation at phase-2. Only C99 belongs to the '*late response*' group (Figure 7). Genes that were activated at phase-1, phase-2 and phase-3 accounted for 71%, 22% and 7% of the total NTF genes in the dataset, respectively.

Genes that were also induced by H_2_O_2 _were more predominant in phase-1 clusters than in phase-2 and phase-3 clusters. Of the twelve phase-1 clusters, only C78 (39%) and C88A (16%) had low proportion of H_2_O_2 _induced genes. Between 50 to 79% of the genes in ten other phase-1 clusters (C79, C83A, C86, C86A, C90A, C91, C94, C97, C97A, C99A) were induced by H_2_O_2 _(Figure 6). In contrast, the proportion of H_2_O_2_-induced genes among phase-2 and phase-3 clusters ranged only between 12 to 49% per cluster (Figure 7). Apparently, genes that were rapidly induced during exposure to chilling (initial 6 hours) are more likely to respond to exogenous H_2_O_2 _than those that were induced at a slower pace (see Additional file 6). These results further support the hypothesis that oxidative burst during the early stages of chilling stress was a primary signal for early gene induction events, and that '*early response*' transcriptome is controlled largely by TFs responding directly to primary oxidative signals. This trend is consistent with the induction of large number of genes associated with disease response, wounding, and ROS scavenging at phase-1, many of which are associated with various signals (ethylene, ABA, auxin, salicylic acid, jasmonic acid) linked directly or indirectly to H_2_O_2_-mediated processes [38,62,69,75].

Promoter elements shared in common by the majority of genes within a cluster were identified to gain further insights on how gene induction is coordinated [7]. Promoter regions (-1,000 to +200) of more than 70% of genes in the total dataset were determined by aligning full-length cDNA with corresponding genomic loci. Delineated promoter regions were used for *ab initio *detection of putative cis-elements by the Dragon Motif Builder algorithms. Motifs with occurrence of 50% or higher in a given cluster were matched with known elements in the TRANSFAC, PLACE and AGRIS databases. Motifs were assigned to specific classes based on known association to specific classes of TFs (Tables 2, 3, 4). The most significantly enriched motifs are related to several classes of elements associated with MYB, bZIP and ERF factors. Motifs associated with TF family were enriched at various levels in 18, 13 and 10 clusters, respectively (Figures 6, 7; Tables 2, 3, 4). WRKY associated motifs were significantly enriched only in 2 clusters. However, some of the possible WRKY target motifs (W-box) were quite similar with the as1/ocs/TGA-like motifs associated with bZIP factors and this may have reduced the occurrence of WRKY-associated cis-elements in the total gene set [31,75]. Motifs associated with bHLH and NAC had low occurrence at the 50% threshold. Other classes of motifs were also detected but could not be assigned to specific TFs (see Additional file 7).

**Table 2:** Enrichment of putative bZIP-target cis-elements in promoters of chilling upregulated genes.

Cluster	Motif	Putative element^a^	Associated class of bZIP (putative)^b^	% (TIC)^c^	e-value
C100A	GGTTTGTA	as1/ocs/TGA-like	Groups D, I, S	71 (10.56)	5e-004

	GAGGAAGA	as1/ocs/TGA-like	Groups D, I, S	69 (13.28)	4e-004

	AAACAATG	as1/ocs/TGA-like	Groups D, I, S	61 (12.36)	9e-005

	GCAATATA	as1/ocs/TGA-like*	Groups D, I, S	58 (11.64)	8e-004

C99	AAATTGATT	as1/ocs/TGA-like	Groups D, I, S	50 (14.12)	3e-004

C99A	TTTTGCTG	as1/ocs/TGA-like	Groups D, I, S	71 (11.97)	7e-004

	TTGGAGAG	RSG element	bZIP	66 (11.78)	3e-004

	CCGTGACA	as1/ocs/TGA-like*	Groups D, I, S	55 (11.52)	6e-004

	TTTGTGTA	as1/ocs/TGA-like*	Groups D, I, S	51 (12.85)	7e-004

C97	ATGTTT(g/t)(a/g)(a/c)	as1/ocs/TGA-like*	Groups D, I, S	62 (12.32)	3e-004

	GCG(a/c/t)ACAA	ABRE-like	Group-A	62 (11.44)	7e-004

	ATATTTTGA	as1/ocs/TGA-like*	Groups D, I, S	54 (13.95)	4e-004

	AGATTAAA	as1/ocs/TGA-like*	Groups D, I, S	52 (12.40)	1e-003

	AATT(a/t)GAG	as1/ocs/TGA-like*	Groups D, I, S	50 (13.45)	8e-004

C97A	CGGCGGCGA	ABRE-like	Group-A	52 (14.65)	9e-004

C94	CGACGACG	as1/ocs/TGA-like	Groups D, I, S	61 (11.77)	1e-004

	GTTTTGAT	as1/ocs/TGA-like	Groups D, I, S	58 (12.33)	8e-004

	GTGATGTG	as1/ocs/TGA-like	Groups D, I, S	53 (12.23)	5e-005

	G_3_TGAC(a/g)A	as1/ocs/TGA-like	Groups D, I, S	52 (11.99)	5e-004

C91	CTCCTCCT	ABRE-like	Group-A	58 (14.14)	9e-004

	CGCCGTTC	Vs1-like	Groups D, I, S	53 (11.37)	2e-004

	AGATGATG	as1/ocs/TGA-like	Groups D, I, S	50 (12.98)	1e-004

	AATTTATG	as1/ocs/TGA-like	Groups D, I, S	50 (12.90)	2e-004

C90A	AATTTGAT	as1/ocs/TGA-like*	Groups D, I, S	56 (12.80)	1e-004

	CGTGGTGT	ABRE-like	Groups D, I, S	51 (11.80)	5e-005

C88A	GGCGGGAG	ABRE-like	Groups D, I, S	53 (12.47)	8e-004

	GGCGGGAG	ABRE-like	Groups D, I, S	53 (12.47)	8e-004

	TCTTCTCTCT	CAMTA3	bZIP	50 (14.80)	5e-004

	GAAAATGA	as1/ocs/TGA-like	Groups D, I, S	50 (12.74)	4e-004

C86	AAACCACA	ABRE-like	Groups D, I, S	69 (11.25)	2e-004

	CAAAAACA	ABRE-like	Groups D, I, S	68 (12.03)	2e-004

	GGCCATCG	as1/ocs/TGA-like	Groups D, I, S	68 (11.35)	2e-004

	AGCGAGAG	ABRE-like	Group-A	61 (11.84)	5e-004

	TACACCAT	ABRE-like	Group-A	60 (11.47)	2e-004

	GAACGATG	ABRE-like	Group-A	57 (11.22)	2e-004

	TCCTCTTCT	CAMTA3/ABRE-like	Groups D, I, S	51 (13.37)	9e-004

	TATATGTA	ABRE-like	Group-A	51 (13.35)	2e-003

	CAAATTGA	as1/ocs/TGA-like*	Groups D, I, S	50 (12.54)	5e-004

C87A	GCGAGGAA	ABRE-like	Group-A	63 (11.21)	2e-004

	AGCAAACAA	ABRE-like	Group-A	51 (13.37)	2e-004

	ACAACGAC	ABRE-like	Group-A	50 (11.91)	2e-004

C86A	CGGTGGCG	ABRE-like	Group-A	70 (12.13)	4e-004

	TGAAGATG	as1/ocs/TGA-like	Groups D, I, S	53 (12.24)	1e-003

	GCTGATTT	as1/ocs/TGA-like	Groups D, I, S	51 (11.92)	5e-004

C83	CTCGCCGC	ABRE-like	Group-A	65 (13.24)	7e-004

	AAATTTGA	as1/ocs/TGA-like*	Groups D, I, S	63 (12.68)	6e-004

	C(c/t)GAGCTC	as1/ocs/TGA-like	Groups D, I, S	57 (11.44)	5e-004

C83A	AATTTGAT	as1/ocs/TGA-like*	Groups D, I, S	70 (12.72)	2e-004

	GGCTC(a/g)A(a/c/g)	as1/ocs/TGA-like*	Groups D, I, S	65 (11.06)	6e-004

	AATTTTGA	as1/ocs/TGA-like*	Groups D, I, S	61 (12.86)	7e-004

	(a/t)GAAATTG	as1/ocs/TGA-like*	Groups D, I, S	53 (11.85)	4e-004

	TCGCCGTC	ABRE-like	Group-A	51 (13.06)	2e-004

	TCTGA(a/t)CA	as1/ocs/TGA-like*	Groups D, I, S	51 (11.80)	7e-004

	GAG(a/g)CGAA	as1/ocs/TGA-like*	Groups D, I, S	51 (13.00)	1e-004

	CCACCCAA	ABRE-like	Group-A	51 (12.44)	7e-004

C81	AGGAGA(a/g)G	as1/ocs/TGA-like	Groups D, I, S	61 (13.03)	1e-004

	GGCCGTG(c/g)	ABRE-like	Group-A	52 (12.65)	2e-004

C79	CGTGGTGT	ABRE-like	Group-A	65 (11.60)	5e-004

	GAAAATGA	as1/ocs/TGA-like	Groups D, I, S	53 (12.04)	2e-004

	ATAATTTGA	as1/ocs/TGA-like	Groups D, I, S	53 (13.74)	9e-004

	ATGTACATTT	as1/ocs/TGA-like	Groups D, I, S	52 (12.82	2e-004

	ATCATGCA	ABRE-like	Group-A	51 (12.69)	4e-004

C78	CTCCTCCT	ABRE-like	Group-A	65 (14.12)	1e-003

	AAATTGATT	as1/ocs/TGA-like*	Groups D, I, S	53 (14.12)	3e-004

^a^Homology with motifs identified in other plant species (TRANSFAC, PLACE and AGRIS databases).

^b^Possible classes of associated bZIP proteins based on recent phylogenetic classification [29] and co-expression.

^c^Percent occurrence in critical promoters relative to background sequences (Total Information Content).

*Also similar to W-box (WRKY) motifs.

**Table 3:** Enrichment of putative ERF/RAV-target cis-elements in promoters of chilling upregulated genes.

Cluster	Motif	Putative element^a^	**Associated class of ERF (putative)**^b^	% (TIC)^c^	e-value
C100A	TTTCTTTG	JA response element-like	Groups VI, VIII, IX	81 (12.14)	7e-004

	ACCTGATAT	rav1b element-like	Groups II, III and RAV1	61 (11.98)	4e-004

	ATTTAGAG	JA response element-like	Groups VI, VIII, IX	60 (11.82)	5e-004

	TATATGAA	JA response element-like	Groups VI, VIII, IX	51 (12.46)	3e-004

C99	CAAACCGA	DRE/CRT-like	Groups II, III and RAV1	62 (11.76)	1e-004

C99A	CCTTCCAT	CArg-box-like	ERF	61 (12.17)	2e-004

	GGCGGCGGC	GCC-box-like	Groups I, IV, VII, X	60 (15.85	1e-004

C97	CGCCGCCG	GCC-box-like	Groups I, IV, VII, X	73 (14.86)	3e-004

C97A	TGAAACAA	rav1b element-like	Groups II, III and RAV1	81 (11.29)	2e-004

C94	TCAAAATT	Ethylene RE-like	ERF	58 (12.32)	1e-004

	GCTCCGCC	GCC-box-like	Groups I, IV, VII, X	53 (12.92)	4e-004

	TATATGAA	JA response element-like	Groups VI, VIII, IX	51 (12.59)	3e-004

	CGCCGCCGC	GCC-box-like	Groups I, IV, VII, X	51 (16.05)	2e-004

C91	GGGCGGCT	GCC-box-like	Groups I, IV, VII, X	54 (12.33)	3e-004

	CCGCCGCCG	GCC-box-like	Groups I, IV, VII, X	50 (16.20)	1e-004

C90A	CGGCGACG	DRE/CRT-like	Groups II, III and RAV1	66 (13.32)	1e-004

	GCCGCCGC	GCC-box-like	Groups I, IV, VII, X	57 (14.28)	2e-004

C88A	CGGCGGCGG	GCC-box-like	Groups I, IV, VII, X	58 (17.37)	3e-005

	CGGCGGCGG	GCC-box-like	Groups I, IV, VII, X	53 (17.37)	3e-005

	CGCCGCCAC	GCC-box-like	Groups I, IV, VII, X	50 (14.54)	3e-004

C87A	CAAGGCCA	GCC-box-like	Groups I, IV, VII, X	57 (11.12)	4e-004

C86	CACCTTTC	rav1b element-like	Groups II, III and RAV1	72 (11.24)	1e-003

	CGCCGCCGC	GCC-box-like	Groups I, IV, VII, X	63 (15.89)	9e-004

C86A	CGTCGGAA	DRE/CRT-like	Groups II, III and RAV1	55 (12.08)	5e-004

C83	GCCGCCGCC	GCC-box-like	Groups I, IV, VII, X	69 (16.31)	3e-004

C83A	GCGT(c/t) GGC	DRE/CRT-like	Groups II, III and RAV1	53 (12.14)	2e-004

C81	GAG(c/g)AGCG	JA response element-like	Groups VI, VIII, IX	69 (12.19)	4e-004

	TCT(a/c)AACA	rav1b element-like	Groups II, III and RAV1	67 (11.43)	6e-004

	GTGG(a/c)GAC	DRE/CRT-like	Groups II, III and RAV1	61 (11.73)	4e-004

	AATTTA(g/t)AG	JA response element-like	Groups VI, VIII, IX	59 (12.45)	3e-004

C80	GGCGGCGGC	GCC-box-like	Groups I, IV, VII, X	60 (16.53)	3e-005

	GAAGCCGA	DRE/CRT-like	Groups II, III and RAV1	53 (12.66)	4e-004

C79	CGCCGCCGC	GCC-box-like	Groups I, IV, VII, X	59 (15.76)	3e-004

	CGGCGACG	DRE/CRT-like	Groups II, III and RAV1	52 (13.85)	4e-004

C78	CAAACCGA	DRE/CRT-like	Groups II, III and RAV1	62 (11.76)	1e-004

^a^Homology with motifs identified in other plant species (TRANSFAC, PLACE and AGRIS databases).

^b^Possible classes of associated ERF/RAV1 proteins based on recent phylogenetic classifications [37] and co-expression.

^c^Percent occurrence in critical promoters relative to background sequences (Total Information Content).

**Table 4:** Enrichment of putative MYB-target cis-elements in promoters of chilling upregulated genes.

Cluster	Motif	Putative element^a^	Associated class of MYB (putative)^b^	% (TIC)^c^	e-value
C100A	AAAACCAT	MYB2-box-like	R2R3-MYB	75 (11.59)	6e-004

	CTTTTGTT	GA response element-like	R1-MYB/R2R3-MYB	60 (12.03)	1e-004

	TGTGGAAG	Pyrimidine box-like	R1-MYB/R2R3-MYB	55 (11.66)	3e-004

	TGTGGAAG	MYB2-box-like	R2R3-MYB	52 (13.26)	6e-005

C99	CATTTGTT	GA response element-like	R1-MYB/R2R3-MYB	64 (12.23)	2e-004

	GCTGTGGT	Pyrimidine box-like	R1-MYB/R2R3-MYB	56 (11.26)	3e-004

C99A	TTTTTTCA	Pyrimidine box-like	R1-MYB/R2R3-MYB	63 (13.88)	3e-004

	G(A_8_)	Pyrimidine box-like	R1-MYB/R2R3-MYB	63 (13.88)	6e-005

C97	(c/t)(A_7_)	Pyrimidine box-like	R1-MYB/R2R3-MYB	73 (16.75)	2e-004

	TAA(T_6_)	Pyrimidine box-like	R1-MYB/R2R3-MYB	67 (14.37)	4e-004

C97A	AAAATCAA	MYB2-box-like	R2R3-MYB	71 (12.93)	4e-004

	AAAGAAAAA	Pyrimidine box-like	R1-MYB/R2R3-MYB	66 (15.13)	2e-004

	ACACAAAC	GA response element-like	R1-MYB/R2R3-MYB	59 (11.48)	2e-004

	TTCTTGCT	GA response element-like	R1-MYB/R2R3-MYB	59 (11.92)	2e-004

	TTTTTTTTAA	Pyrimidine box-like	R1-MYB/R2R3-MYB	59 (16.60)	2e-005

	TTGTTTTGT	GA response element-like	R1-MYB/R2R3-MYB	57 (12.93)	3e-004

	ATTTAACA	GA response element-like	R1-MYB/R2R3-MYB	55 (11.99)	3e-004

	GCTGCTGC	GA response element-like	R1-MYB/R2R3-MYB	50 (12.99)	8e-004

C94	TAGTTTTT	MYB1-box-like	R2R3-MYB	70 (11.74)	2e-004

	CTCCCTCCG	GA response element-like	R1-MYB/R2R3-MYB	57 (13.64)	2e-004

	AGCTGGAG	GA response element-like	R1-MYB/R2R3-MYB	55 (12.00)	9e-005

	AGCATTTG	GA response element-like	R1-MYB/R2R3-MYB	51 (12.29)	5e-004

C91	TTCTTTTTTT	Pyrimidine box-like	R1-MYB/R2R3-MYB	58 (15.52)	4e-004

	TTAGGGTTT	MYB2-box-like	R2R3-MYB	58 (12.70)	2e-004

C90A	TTTTTTCA	Pyrimidine box-like	R1-MYB/R2R3-MYB	63 (13.45)	4e-004

	AAATCCAA	GA response element-like	R1-MYB/R2R3-MYB	57 (12.72)	1e-003

	TTAGTTTAT	MYB1-box-like	R2R3-MYB	52 (12.89)	2e-004

	TTGTTTTT	GA response element-like	R1-MYB/R2R3-MYB	51 (13.14)	2e-004

C88A	GAAACCAT	MYB2-box-like	R2R3-MYB	78 (11.18)	1e-004

	CGGTGGAT	MCB1/MCB2-like	R2R3-MYB	73 (11.07)	1e-004

	TTTTTTCA	Pyrimidine box-like	R1-MYB/R2R3-MYB	69 (13.14	6e-004

	AAACCCAA	GA response element-like	R1-MYB/R2R3-MYB	63 (12.32	2e-004

	GGGGATCG	MYB2-box-like	R2R3-MYB	58 (11.82)	1e-004

	GAAACCAT	MYB2-box-like	R2R3-MYB	58 (11.95)	1e-004

	GTGATTAGC	MYB2-box-like	R2R3-MYB	55 (11.32)	9e-004

	G(T_8_)	Pyrimidine box-like	R1-MYB/R2R3-MYB	52 (14.78)	7e-004

	AGCTGGAG	GA response element-like	R1-MYB/R2R3-MYB	50 (11.89)	1e-004

C87A	TAAGTTTT	Pyrimidine box-like	R1-MYB/R2R3-MYB	65 (12.65)	8e-004

	AACAATTT	GA response element-like	R1-MYB/R2R3-MYB	63 (11.80)	5e-004

	AAACCATG	MYB2-box-like	R2R3-MYB	61 (12.27)	8e-004

	GTTTTTTTT	Pyrimidine box-like	R1-MYB/R2R3-MYB	54 (15.01)	4e-004

	ATATTTCC	Pyrimidine box-like	R1-MYB/R2R3-MYB	50 (12.51)	6e-004

C86	AAATCCAA	MYB2-box-like	R2R3-MYB	60 (12.33)	3e-004

	AACCATGG	MYB2-box-like	R2R3-MYB	60 (11.10)	3e-004

C86A	TTTTTTCA	Pyrimidine box-like	R1-MYB/R2R3-MYB	72 (13.48)	3e-004

	TTTTTATT	Pyrimidine box-like	R1-MYB/R2R3-MYB	68 (14.18)	2e-004

	ATCATTTT	Pyrimidine box-like	R1-MYB/R2R3-MYB	62 (12.27)	7e-004

	TCCCTTTT	Pyrimidine box-like	R1-MYB/R2R3-MYB	57 (13.18)	7e-004

	ACATTTTT	Pyrimidine box-like	R1-MYB/R2R3-MYB	50 (13.18)	2e-004

C83	GGAAAAAA	Pyrimidine box-like	R1-MYB/R2R3-MYB	67 (13.45)	4e-004

C83A	GAAAAAGG	Pyrimidine box-like	R1-MYB/R2R3-MYB	61 (11.91)	4e-004

C80	AAATCCTT	GA response element-like	R1-MYB/R2R3-MYB	72 (11.89)	8e-005

	TTTTTTCA	Pyrimidine box-like	R1-MYB/R2R3-MYB	55 (13.28)	4e-004

	TTTTCAAT	Pyrimidine box-like	R1-MYB/R2R3-MYB	53 (12.23)	2e-003

C81	(c/g)(A_9_)	Pyrimidine box-like	R1-MYB/R2R3-MYB	68 (15.62)	3e-004

'	GG(A_6_)	Pyrimidine box-like	R1-MYB/R2R3-MYB	64 (13.23)	4e-004

	(c/g)AT(A_5_)	Pyrimidine box-like	R1-MYB/R2R3-MYB	61 (12.19)	3e-004

	ATATTTTC	Pyrimidine box-like	R1-MYB/R2R3-MYB	54 (12.51)	4e-004

C79	TTTTTTCA	Pyrimidine box-like	R1-MYB/R2R3-MYB	65 (13.14)	2e-003

	GTGATATC	Pyrimidine box-like	R1-MYB/R2R3-MYB	62 (11.12)	6e-004

	CATCTTTT	GA response element-like	R1-MYB/R2R3-MYB	53 (12.53)	3e-004

	ATTAAATTA	Pyrimidine box-like	R1-MYB/R2R3-MYB	53 (13.65)	3e-004

C78	TTAGGTTTT	MYB2-box-like	R2R3-MYB	68 (12.28)	4e-004

	CATTTGTT	GA response element-like	R1-MYB/R2R3-MYB	64 (12.23)	2e-004

	GCTGTGGT	Pyrimidine box-like	R1-MYB/R2R3-MYB	56 (11.26)	3e-004

^a^Homology with motifs identified in other plant species (TRANSFAC, PLACE and AGRIS databases).

^b^Possible classes of associated MYB proteins based on recent phylogenetic classifications [50] and co-expression.

^c^Percent occurrence in critical promoters relative to background sequences (Total Information Content).

**Figure 8:**
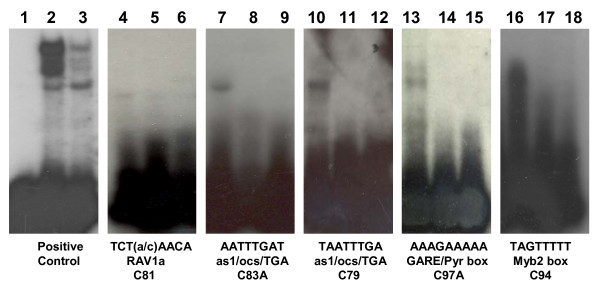
**Analysis of *in vitro *binding between a select group of putative promoter cis-elements and nuclear proteins extracted from chilling stressed rice seedlings**. Lanes 1, 6, 9, 12, 15, 18 = oligonucleotide minus nuclear extract; Lanes 2, 4, 7, 10, 13, 16 = oligonucleotide plus nuclear extract; Lane 3 = positive control oligonucleotide plus unlabeled competitor plus nuclear extract; Lanes 5, 8, 11, 14, 17 = oligonucleotide plus unlabeled competitor plus nuclear extract. Data represent the results of 2 repetitions for each motif species.

The predominance of MYB, bZIP and ERF associated motifs were also quite apparent when the enrichment analysis was performed on the entire dataset (2,456 genes) as a single group (without clustering) and compared to a subset of known genes not involved in stress response (background control). This result indicated that enrichment was not due to random occurrence of frequently occurring sequences in the japonica rice genome. To assess the biological significance of *ab initio *prediction, a select group of putative cis-elements from the major clusters were tested for binding with nuclear proteins *in vitro *by electrophoretic mobility shift assay (EMSA). The rav1-like (TCT(a/c)AACA), as1/ocs/TGA-like (AATTTGAT, TAATTTGA), pyrimidine box-like (AAAGAAAAA) and MYB2-like (TAGTTTTT) motifs enriched in C81, C83A, C79, C97A and C94, respectively, showed band shifts in the presence of pooled nuclear proteins from chilling stressed (10°C) seedlings (Figure 8). These results indicate that the sequences are *bona fide *binding sites of low temperature-induced nuclear proteins.

### bZIP regulatory clusters

Motifs of the as1/ocs/TGA-like and ABRE-like classes were the most significantly enriched among the possible bZIP-target sequences [13,75,76]. Occurrence of the as1/ocs/TGA-like class was particularly prominent (score = 100 to 350) in C79, C83, C83A, C86, C86A, C90A, C91, C94, C97, C99A and C100A (Figures 6, 7; Table 2). Only two of these clusters (C83, C100A) were activated at phase-2 and the rest were activated at phase-1, indicating that as1/ocs/TGA-like motifs are critical features of rapid response genes. Clusters enriched with as1/ocs/TGA-like elements also appeared to be more responsive to exogenous H_2_O_2 _based on high proportion (53 to 79%) of H_2_O_2_-induced genes among those clusters. This is an indication of direct relationships between rapid induction, as1/ocs/TGA-like element and responsiveness to H_2_O_2_, which we also observed in our previous analysis of a smaller subset of upregulated genes [17]. The as1/ocs/TGA elements are key components of regulatory modules involved in auxin, salicylic acid and jasmonic acid mediated gene expression in response to wounding, oxidative stress and pathogen attack, providing direct support that '*early response*' genes are direct consequences of primary oxidative signals [28,30,31,33,68,77]. This is also in agreement with the timing of H_2_O_2 _spikes within the initial 6 to 12 hours at 10°C (Figure 5).

Activation of as1/ocs/TGA-like element-enriched clusters can be associated with several phase-1 (Os08g43090: *RF2b*-like protein, Os06g41100: *TGA10*, Os08g38020: *AtbZIP148*-like protein) and a phase-2 (Os04g10260: bZIP domain protein) bZIP genes that were also induced by H_2_O_2 _(Figure 2). In tobacco, *TGA10 *has been shown to directly bind to an as1/ocs/TGA-like sequence and its transient expression enhanced the expression of downstream pathogenesis-related (PR) target genes via auxin, salicylic acid and jasmonic acid signaling pathways [31]. Consistent with this, our recent results showed that as1/ocs/TGA-like element-containing genes were among those that were activated in a chilling or H_2_O_2_-independent manner in transgenic rice overexpressing *TGA10 *(data not shown). In addition, three other H_2_O_2_-induced bZIP genes that were activated at phase-3 (Os02g09830/Os02g03960: ocs-binding factor, Os05g37170: *TGA6*) are also likely to be involved in the regulation of as1/ocs/TGA-like element-enriched clusters. These genes could be the direct targets of bZIP genes induced at phase-1, thus they are possible components of ROS-bZIP sub-regulons. The ROS-bZIP genes belong to the groups involved in defenses to pathogens, photomorphogenesis, GA response and sucrose signaling, and this suggests important roles of their downstream regulon in the integration of stress and developmental responses [24,29].

ABRE-like motifs typically found among ABA-regulated genes were particularly enriched (scores = 100 to 400) in C88A, C91, C94 and C99A (Figure 6; Table 2) [5,13,75]. These clusters are likely due at least in part to two ABRE-associated TFs induced at phase-3 (Os02g52780: *AB15*, Os01g64000: *ABF*). In addition to ABRE-like motifs, C91, C94, and C99A were also enriched with as1/ocs/TGA-like elements and all three clusters had large proportions of H_2_O_2 _induced genes (67%, 70%, 64%, respectively). These clusters were activated in two phases (early and late), which are likely due to early and late activities of bZIP-TGA and bZIP-ABF genes, respectively (Figure 2). WRKY factors appear to be involved in modulation of ABA-mediated expression, particularly in C91 which is enriched with W-box-like motifs. Some of the as1/ocs/TGA-like motifs in C94 and C99A were also quite similar to W-box-like elements, which could be functioning as WRKY target elements [78]. WRKY genes could be regulatory fine-tuners by acting as repressors of ABA-induced genes at phase-1 and phase-2 [60].

### ERF regulatory clusters

Enrichment of ERF associated motifs in several clusters was consistent with the activities of several ERF genes belonging to groups I, II, III, IV, VI, VII, VIII, IX and X [37]. ERF associated motifs were divided into two classes. The first includes DRE/CRT-like and rav1-like motifs, which are typically found among abiotic stress induced genes controlled by *DREB/CBF*, *RAV1 *and *RAP2 *[3,5,6,27]. This class of motifs occurred in 10 clusters but their enrichment was relatively subtle compared to other non-ERF-type motif classes (Figures 6, 7; Table 3).

Clusters C78, C81, C86, C97A and C90A were most enriched with DRE/CRT-like and rav1-like elements (scores = 61 to 130), while C79, C80, C83A, C86 and C99 had intermediate (scores = 50 to 60) enrichment (Figures 6, 7). Clusters C78, C79, C80, C83A, C86, C90A and C97A were activated at phase-1 and this is likely due to several Group-II (Os06g11940: *RAP2.1/2.6/2.10 *group), Group-III (Os02g45450: *DREB1A/CBF3*, Os08g43210/Os09g35010: *DREB1B/CBF1*, Os01g73770: *DREB1C/CBF2*), Group-VII (Os12g40960: AP2 protein, Os03g08500: ERF2-like) and RAV1-like (Os01g04750: *ABI3/VP1*) genes that were also activated at phase-1 (Figure 2). Clusters C80 and C81 were activated at phase-2, which are likely due to a different set of Group-II (Os03g15660: *RAP2.1/2.6/2.10 *group), Group-III (Os06g03670: *DREB1A/CBF3*) and Group-VII (Os03g08490: *ERF2*-like) genes distinct from those acting on phase-1 genes. Two genes from Group-III (Os09g35020: *DREB1D/CBF3*) and RAV1-family (Os01g04800: *ABI3/VP1*) appear to be involved in '*late response*' regulation in C99. Many *DREB/CBF*-regulated genes are also activated through ABA-mediated pathway [5]. Thus, the Group-I (Os04g44670: *AtERF053*, Os06g11860: *AtERF058*, Os03g09170: *AtERF058*), Group-Group-IV (Os05g28350: *AtERF052/ABI4*) and Group-X (Os09g28440) genes associated with ABA response may also be involved in the activation of DRE/CRT/rav1-like element-enriched clusters in response to ABA.

The second group of ERF associated elements includes the GCC-box-like and jasmonic acid response element-like (JAre) motifs (Figures 6, 7; Table 3) involved in disease and wounding responses and regulation of indole alkaloid metabolism [37-39]. These motif classes were particularly enriched in C81, C83, C88A, C91, C94 and C100A. Clusters C88A, C91 and C94 were activated at phase-1 and this can be attributed to a number of group-VIII (Os04g52090: *AtERF4*, Os03g08500: *AtERF2*, Os06g47590/Os04g57340: *AtERF3*) and group-IX (Os08g44960: *AtERF15*, Os09g39810: *PTI5/AtERF14*) genes. Another gene from Group-VIII (Os05g41780: *AtERF4*) likely contributed to the activation of C81, C83 and C100A (Figure 3).

ERF genes are also likely components of oxidative-mediated regulatory mechanism. Genes induced by H_2_O_2 _belong to Group-I (Os06g11860/Os03g09170: *AtERF058*, Os04g44670: *AtERF053*), Group-II (Os06g11940: *RAP2.1/2.9/2.10 *group), Group-III (Os01g73770: *DREB1C/CBF2*, Os09g35020: *DREB1D/CBF4*), Group-IV (Os05g28350: AtERF052/ABI4), Group-VII (Os12g40960: AP2 protein, Os03g08490: *ERF2*) and Group-X (Os09g28440: B-3 group). Possible targets of these TFs are in C94, C91 and C100A. These clusters were particularly enriched with H_2_O_2_-induced genes and had significant enrichment of GCC-box/JAre-like and/or DRE/CRT/rav1-like motifs (Figures 6, 7).

### MYB regulatory clusters

Potential MYB-target sequences were ubiquitous in almost all clusters implying that MYB genes are functioning either singly or in synergy with other TFs (Figures 6, 7; Table 4). MYB-related motifs are quite diverse, but can be classified into two classes. MYB2-box-like motifs were significantly over-represented (scores = 50 to 250) in C79, C86, C87A, C88A, C90A, C91, C94, C97A and C100A (Figures 6, 7; Table 4). Their high occurrence among chilling induced genes appears to be linked to the activities of R2R3-type MYB, which have been shown to directly interact with MYB2-box-like elements in the promoters of osmotic, drought and ABA induced genes [50,52,79-81]. Several Os*MYB2 *(Os01g18240, Os05g04820) and Os*MYB4 *(Os04g43680, Os02g41510, Os10g33810) homologs are likely the major players in the activation of C79, C86, C87A, C88A, C90A, C91, C94, C97A and C100A, perhaps in response to ABA, H_2_O_2 _or both. The second group of MYB-target elements includes several species of GA response element-like (GARE) and pyrimidine-box-like motifs, which are typically found among gibberellic acid (GA) regulated genes [79]. These elements were particularly enriched (scores = 60 to 500) in C78, C79, C80, C81, C83, C83A, C86A, C87A, C88A, C90A, C91, C94, C97, C97A, C99, C99A and C100A. Recent studies have shown that R1-MYB particularly the SHAQKYF-type activates their GA-responsive targets through GARE and pyrimidine box-like elements [52].

R1-MYB genes are generally involved in seedling development and growth and they are known to regulate genes encoding hydrolytic enzymes involved in carbohydrate breakdown (amylases, glucanases, carboxypeptidases) for energy maintenance during early seedling growth [82,83]. Consistent with this, a number of R1-MYB genes were induced at 10°C including few SHAQKYF-type (Os06g07640, Os08g04840). Activities of these and other related R1-MYB genes are likely responsible for activating the GARE or pyrimidine box-like element-enriched clusters. Seedlings at the early stages of growth (V_3_) were used in the microarray studies, during which the endosperm was only partially degenerated. Growth at this stage requires mechanisms to integrate stress and developmental signals. The GARE or pyrimidine-box-like element-enriched clusters may be important for the integration of early growth and stress-related energy demands, which have been suggested as the key aspects of seedling vigor expression [84].

Broad distribution of MYB-related elements among chilling upregulated genes has important regulatory and physiological implications. MYB-target elements often occur in combination with one or more other types of element (Figures 6, 7), which implies that MYB factors are key components of combinatorial control mechanisms in conjunction with other major classes of chilling induced TFs (bZIP, ERF, WRKY) either as activators or repressors [49,50]. MYB-regulatory clusters may be involved in both stress and growth related processes. Within the context of seedling cold tolerance, MYB factors may be involved in coordinating the expression of genes required to modulate growth related processes at sub-optimal temperature.

### Combinatorial control of chilling stress transcriptional network: Hypothetical models

**Figure 9:**
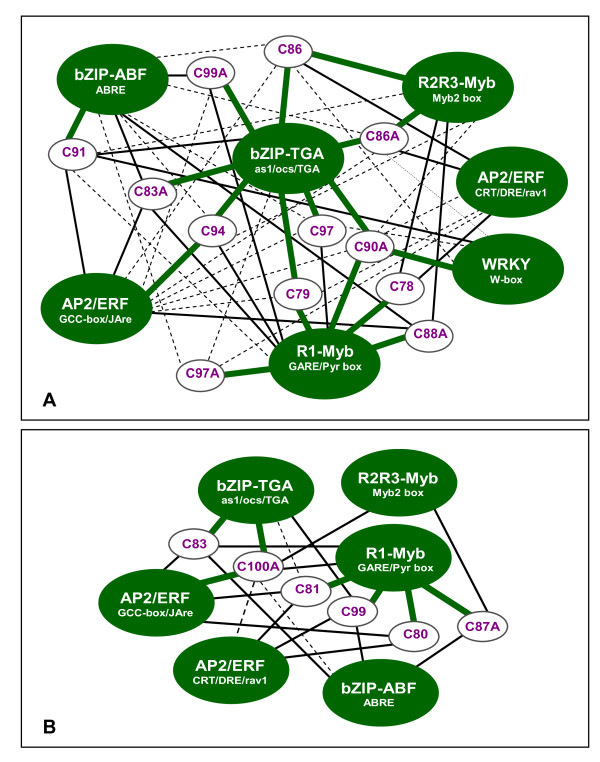
**Hypothetical models of chilling stress transcriptional regulatory network**. (A) phase-1 clusters; (B) phase-2 and phase-3 clusters. Models were based on integrative analysis of temporal co-expression, promoter motif enrichment and responses to oxidative mimic (see Figures 6 and 7). Each expression cluster is connected with all possible transcriptional regulator(s) based on coordinate expression patterns and over-representation of the target cis-elements associated with that class of transcription factor. Heavy green line represents the primary regulatory mechanism (most enriched elements). Solid black and dotted gray lines represent secondary and tertiary regulatory mechanisms, respectively.

Our current data indicate that regulatory modules involving bZIP Group-D, Group-I and Group-S (as1/ocs/TGA-like clusters), bZIP Group-A (ABRE-like clusters), *DREB/CBF, RAP2 *and *RAV1 *(DRE/CRT/rav1-like clusters), ERF (GCC box/JAre-like clusters), R1-MYB (GARE/pyrimidine-box-like clusters), R2R3-MYB (MYB2-box-like clusters), and WRKY (W-box-like clusters) TFs define the overall chilling stress response regulome of japonica rice. Based on these information, hypothetical models depicting the integration of various network components were established reflecting the complexity and hierarchical interaction among the various TFs acting on a given subset of chilling induced genes (Figure 9). Based on the cis-element enrichment trends shown in Figures 6 and 7, some clusters involve relatively simple activation mechanism with one dominant class of regulatory genes. Examples are C83A, C91, C97A and C99A, which were primarily controlled by as1/ocs/TGA (bZIP), ABRE (bZIP), GARE/pyrimidine box (R2R3-MYB) and as1/ocs/TGA (bZIP) regulatory modules, respectively. Other clusters involved complex mechanisms with two or more dominant classes of TFs that may either be activated independent of each other or functioning as components of combinatorial control mechanisms. Examples are C79, C86, C90A and C100A, which involved as1/ocs/TGA (bZIP) + GARE/pyrimidine box (R2R3-MYB), as1/ocs/TGA (bZIP) + Myb2-box (R2R3-MYB), as1/ocs/TGA (bZIP) + GARE/pyrimidine box (R2R3-MYB) + W-box (WRKY), and as1/ocs/TGA (bZIP) + GCC-box/JAre (ERF), respectively. Many examples of the combinatorial control are well known particularly those involved in ABA responses [26,77,85,86]. At the center of the chilling stress regulatory network is ROS (H_2_O_2_) which acts as primary signal triggering the activities of '*early response*' expression clusters. For instance, as1/ocs/TGA-like motifs are dominant in C83A and this cluster was also enriched with H_2_O_2_-responsive genes (65%). Therefore, an oxidative signal-mediated bZIP-TGA regulon appears to be a major component of this cluster. Similarly, C97A appeared to be regulated by R1-MYB. This cluster only had moderate enrichment of H_2_O_2_-responsive genes (Figure 6), thus an oxidative-independent regulon is likely to be part of this cluster.

Many of the putative ERF-regulated clusters represent examples of complex regulatory mechanisms (Figure 9). Clusters C81 and C86 for instance are enriched with DRE/CRT-like or rav1-like motifs and one or more other class of elements. Both clusters have low proportion of H_2_O_2_-responsive genes (Figures 6, 7), thus *DREB/CBF *regulon is likely to be a component of these clusters. Genes belonging to these clusters are likely to be regulated by combinatorial mechanism involving other TFs such as MYB for C81 and bZIP-TGA and MYB for C86 (Figure 9). Enrichment of DRE/CRT-like and rav1-like elements was less pronounced compared to other types of elements (Figures 6, 7, 9). It is possible that milder cold stress activates only a fraction of the entire regulon that would otherwise be induced under more severe cold conditions such as those that induce CA in temperate plants. It has been suggested that differences among plant species with respect to *DREB/CBF *regulon could be due to limited distribution of DRE/CRT-like elements in the genomes of chilling sensitive species and this might be true in rice [42].

Unlike bZIP, ERF and MYB-related elements, motifs associated with WRKY, NAC and bHLH TFs had lower enrichment among the chilling induced genes (Figures 6, 7; see Additional file 7). W-box-like enrichment in C90A appears to be associated with the function of WRKY factors as repressor of ABA-inducible expression. Preliminary analysis of chilling downregulated genes indicate that putative WRKY, MYC and NAC-target elements were among the enriched motifs (data not shown). Thus, most WRKY, NAC and MYC genes are likely functioning primarily as repressors of chilling stress response gene expression [54,59,60,87].

### Physiological implications of upregulated transcriptional clusters

Of the 2,456 chilling upregulated NTF genes, 93% (2,295) could be assigned to one or more molecular or biochemical process based on integrated analysis of Gene Ontology [21] and protein domain i.e., INTERPRO [88] databases (see Additional file 5). Nearly 30% (699) were described with various types of '*cellular response' *or '*stress*' related keywords including defense (GO:0006952), stress (GO:0006950), water (GO:0009725), wounding (GO:0009611), oxidative (GO:0006979), cold (GO:0050826, GO:0042309, GO:0050825) and various stimuli including biotic (GO:0009607), abiotic (GO:0009628), external (GO:0009605) and hormone (GO:0009725). Three functional categories reflecting the major processes involved in chilling stress response mechanism were established.

### Stress signaling and response regulation

**Figure 10:**
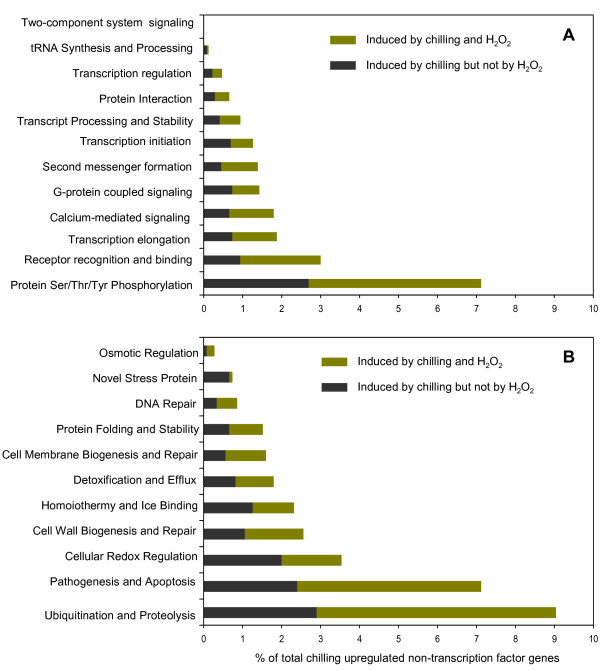
**Representation of various functional categories (molecular and/or biological processes) among the chilling stress upregulated genes**. (A) Signaling and response regulation; (B) Cellular defense and rescue. Also see Additional files 8, 9 and 10 for specific GO/IPRO terminologies relevant to each functional category.

Processing and transduction of environmental signals to the nucleus constitute the initial events in the execution of adaptive responses [4,7,62]. About 20% (494) of the upregulated NTF genes are associated with signaling and response regulatory processes such as protein phosphorylation (GO:0004674, GO:0004713), receptor recognition (GO:0004872, GO:0007154, GO:0004871), Ca^2+ ^signaling (GO:0005509, GO:0005544), small GTPase coupled signaling (GO:0007186, GO:0007264), and generation of second messengers (GO:0046488, GO:0007165, GO:0004435) (Figure 10; see Additional file 5).

A significant increase in the rate of mRNA synthesis was implied by genes required for the assembly of basal transcription machinery including those involved in transcription initiation and elongation, tRNA synthesis, and transcript processing (GO:0003700, GO:0003723, GO:0003712, GO:0016439, GO:0003677, GO:0003899, GO:0030528, GO:0003702). Enhanced activities of these genes reflect an increased rate of *de novo *mRNA synthesis required for activation of response-related transcriptome. Nearly 60% of genes associated with signaling and response regulation were also induced by H_2_O_2_, further supporting the central role of oxidative signaling in chilling stress response mechanism. Although the genes in this category are widely distributed among 18 clusters, phase-1 clusters tend to be more enriched than phase-2 and phase-3 clusters, further indicating that critical signaling events occur during the initial 6 hours.

### Cellular defense and rescue

About 30% (769) of total NTF genes are associated with cellular defense and rescue processes. Genes involved in biogenesis or repair of damaged cellular components comprised about 14% of total NTF genes (Figure 10; see Additional file 5). Dominant classes were chaperones, heat shock proteins and proteases involved in rescue or turnover of damaged proteins (GO:0006508, GO:0006511, GO:0016567, GO:0000151, GO:0030693, GO:0004185), proteins involved in membrane and cell wall biogenesis (GO:0008654, GO:0045300, GO:0006869, GO:0042546, GO:0042545) and DNA repair (GO:0006284, GO:0006281, GO:0003684) [89].

Genes associated with oxidative stress including cellular redox regulators (GO:0045454, GO:0004602, GO:0004601, GO:0006801, GO:0006979; see Additional file 8) and pathogen defense and apoptosis-related proteins (GO:0009607, GO:0009605, GO:0009405, GO:0004568, GO:0006952, GO:0006915, GO:0042742; see Additional file 9) represent another major class of defense and rescue associated genes with about 11% of total NTF genes. Cellular redox regulatory processes include the components of glutathione system, respiratory burst proteins, and ROS-scavengers such as peroxidase, superoxide dismutase, catalase, ascorbate peroxidase, thioredoxins, and metallothionein-like proteins [73]. Genes associated with defenses against pathogens include a large number of NBS-LRR-type disease resistance proteins and various pathogenesis-related (PR) proteins such as chitinases, glucanases, thaumatin-like, germin-like, cupin-like, and dirigent proteins, viral, bacterial, and elicitor-induced proteins, and proteins involved in phenylpropanoid, salicylic acid, jasmonic acid and phytoallexin metabolism [90-94]. This trend shows striking similarities between chilling-induced defenses and those associated with responses to pathogens and wounding, all of which are linked to H_2_O_2 _signaling [75,95].

About 2% of genes associated with cellular defense and rescue are involved in detoxification and efflux processes (GO:0006855, GO:0015904, GO:0006904, GO:0006887, GO:0006814) (Figure 10; see Additional file 10). Various classes of multi-drug resistance, MATE efflux and other types of proteins involved in the extrusion or sub-cellular sequestration of toxic compounds were in this group [96,97]. Many of these genes are also involved in defenses against pathogens. Based on the expression of osmotins and aquaporins and other genes involved in galactinol and trehalose biosynthesis, maintenance of root and leaf water balance and cellular osmotic adjustment (GO:0005992, GO:0009719; see Additional file 5) are important components of defense mechanism [98-100]. Genes associated with thermal regulation such as homoiothermic/freezing-associated proteins (GO:0042309, GO:0050825, GO:0050826) were also induced with more than 2% of total NTF genes (see Additional file 5).

A small group of novel stress-induced genes were also assumed to be part of the defense and rescue category, with nearly 1% of total NTF genes. These genes are known only by virtue of their responsiveness to developmental and environmental stress factors such as cold, drought, salinity, wounding, hypoxia, ABA and senescence (see Additional file 5). Included in this group are several homologs of stress-related genes belonging to the early responsive to dehydration (ERD) and LEA/dehydrin families.

Nearly 60% of genes associated with cellular defense and rescue mechanism were induced by both chilling and H_2_O_2_. Many involved in redox regulation, pathogen defense, apoptosis and proteolysis (Figure 10). This trend implies that defense processes are essentially consequences of chilling-induced oxidative stress and this is consistent with the fact that abiotic and biotic stresses induce a common set of genes through an oxidative-mediated pathway. Cellular defense and rescue related genes are widely distributed in all of 18 clusters. However, phase-1 clusters tend to be more enriched with this functional category than phase-2 and phase-3 clusters (see Additional file 6) implying that short-term adaptation depends on timely activation of defense and rescue mechanisms.

### Physiological adjustment and sustenance

Maintenance of cellular homeostasis is critical for sustained growth and survival under sub-optimal temperature. Cellular physiology must be adjusted to accommodate stress-related demands and metabolic requirements. Means to compensate or replenish metabolic intermediates that have been diverted towards costly defense and rescue processes are necessary for physiological sustenance. The largest category of NTF genes represents diverse molecular and biochemical functions suggesting the nature of physiological adjustment and sustenance processes. This category accounts for more than 40% (1,032) of the total NTF genes (see Additional files 5, 11).

Increase in *de novo *protein synthesis was indicated by genes involved in ribosome assembly and translation (GO:0006412, GO:0003735, GO:0008135, GO:0006446) and protein folding and modification (GO:0006464, GO:0019538, GO:0006457). Genes involved with cellular energetics (GO:0006118, GO:0006096, GO:0006094, GO:0015979, GO:0006091), transport mechanism and facilitation (GO:0006810, GO:0005215, GO:0006865, GO:0015986, GO:0016469, GO:0008643, GO:0046907), carbohydrate, lipid, amino acid and secondary metabolic processes (GO:0008610, GO:0006633, GO:0008299, GO:0006629, GO:0009058, GO:0005975, GO:0019748, GO:0006807), cellular biogenesis (GO:0005875, GO:0005856) and growth (GO:0006260, GO:0016575, GO:0006333, GO:0006334) were highly represented in this category. Genes associated with physiological adjustment and sustenance are widely distributed among 18 expression clusters but more highly represented in the slower clusters (phase-2, phase-3). This trend suggests that physiological adjustment and sustenance processes occurred largely during the middle to later stages of stress (see Additional file 6) and reflects the biochemical costs of regulatory and defense related activities during the earlier stages of stress. It also suggests that '*late response*' genes are important for recovery process.

### Agronomic implications of chilling stress regulatory clusters

Stress tolerance potential is governed by quantitative trait loci (QTL) and TFs are likely to be major components of QTL. To investigate possible association between stress tolerance QTL and the major activators of oxidative-mediated chilling stress response, genomic locations of chilling upregulated TFs were determined in relation to genomic boundaries of relevant QTL that have been anchored to the Nipponbare genome sequence [21]. The ratio of QTL-associated to non-QTL-associated genes in the total annotated gene set in the microarray (whole genome) was determined by Fisher exact test and compared with the corresponding ratios within the upregulated gene subset. Significant enrichment was defined by a higher ratio in the upregulated gene subset (relative to genome-wide ratio) at p < 0.25. A total of 42 chilling induced TFs were located within the genomic boundaries 46 QTL for cold tolerance (COLDTL), seedling vigor (SDLVIG), osmotic adjustment capacity (OSADJCAP), salt sensitivity (SALTSN), chlorophyll content (CHLCN), leaf rolling (LFRL), and speed of germination (GERMSP) (see Additional file 12). SDLVG, CHLCN, COLDTL and OSADJCAP had the most number of co-localized TFs with 24, 16, 12, and 10 genes, respectively.

**Figure 11:**
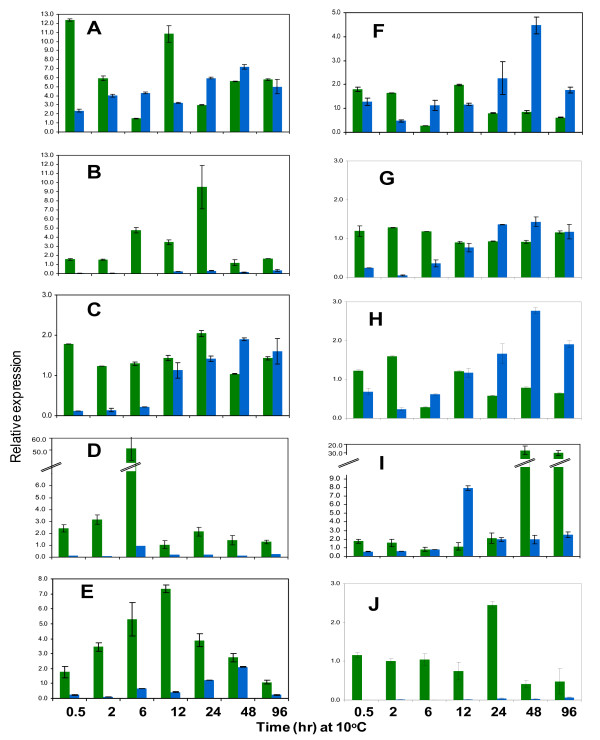
**Differential expression of chilling induced genes in Nipponbare (less sensitive) and INAP12 (more sensitive)**. Transcript levels were compared by quantitative real time PCR. Cultivars can be distinguished by the relative speed of gene induction. In general, induction occurs earlier and was more robust in Nipponbare (green) than INIAP12 (blue). Expression analysis was based on the average of three replicates normalized against a constitutively expressed actin gene. a = Os08g43090 (*RF2b*-like protein); b = Os06g41100 (*TGA10*); c = Os08g38020 (*AtbZIP148*-like protein); d = Os08g43210 (*DREB1B/CBF1*); e = Os09g35020 (*DREB1D/CBF4*); f = Os02g41510 (*OsMyb4*); g = Os06g13460 (jasmonate-induced O-methyltransferase); h = Os10g36270 (NBS-LRR protein); i = Os02g43790 (ethylene responsive protein); j = Os05g14260 (peroxidase-29).

**Figure 12:**
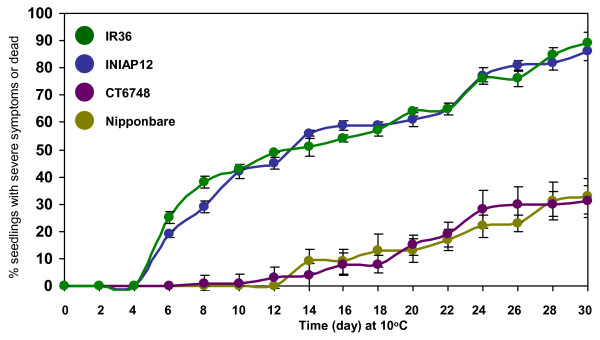
**Seedling survival of japonica (Nipponbare, CT6748) and indica (INIAP12, IR36) rice cultivars**. Seedlings were first germinated at optimum temperature (28°C) before exposure to constant 10°C day/night temperature regime. Injury symptoms were based on Standard Evaluation System, where a score of 1 = seedlings are dark green and healthy, 3= seedlings are pale green, 5 = seedlings are yellow and partially withering, 7 = seedlings are brown and withered, and 9 = seedlings are dead. Occurrence of seedlings with a score of ≥3 was monitored for a 30-day period. A total of 120 plants were analyzed for each cultivar. Values represent the average of three replicates (SE shown as error bars).

We also examined the differences in expression of representative TFs between Nipponbare and INIAP12 rice cultivars (Figure 11). These cultivars represent contrasting sensitivity to chilling based on seedling survival test (Figure 12). Chilling-induced expression of TFs in Nipponbare occurred earlier and was more robust than INIAP12, consistent with results from previous studies [14,17]. Differential expression of TFs positively correlates with the responses of japonica and indica cultivars to chilling. About 4 weeks after continuous exposure to 10°C, 50% of indica seedlings were either dead or with severe injury symptoms. Mild injuries occurred in japonica seedlings only after about 6 weeks. Based on these results, stress response transcriptome is tightly associated with the expression of vigor.

## Conclusion

We studied the regulatory and physiological implications of chilling stress (10°C) transcriptome of japonica rice based on integrative analysis of whole-genome expression profiles and promoter architectures. Our analysis has taken into consideration the hypothesis that oxidative signaling is central in integrating various components of the regulatory network. We addressed this hypothesis by dissecting the commonalities between a direct response to cold stimulus and responses to low temperature-independent mimic of oxidative signaling. From the systems-level approach employed in this study, several important themes emerged. First, oxidative signaling by H_2_O_2 _is at the center of the regulatory network, particularly in relation to the execution of '*early response*' mechanisms (initial 24 hours). H_2_O_2 _has long been recognized as a major trigger of stress response signaling and its important role in interfacing abiotic and biotic stress responses with growth and development has been established [67,68,101,102]. Until now, direct links of the primary oxidative signals to chilling-associated transcriptional changes has not been demonstrated at the genome-wide scale. Our current results contribute important information that fills this knowledge gap.

About 60% of chilling stress induced genes are triggered by oxidative signals either as primary or secondary targets. Much of the oxidative-mediated changes in gene expression occurred during the initial 24 hours and the composition of the transcriptome has striking similarities to disease response mechanisms in terms of the activities of genes involved in redox regulation, responses to organic toxins and wounding, phenylpropanoid and indole alkaloid metabolism, and jasmonic acid, salicylic acid and ethylene signaling [39,62,73]. H_2_O_2 _is likely to be the primary signal closest to the original stimulus (chilling) providing the initial trigger for a cascade of molecular processes leading to an intricately interconnected gene expression circuitry [101,102]. The timing and scope of oxidative mediated regulatory clusters justify their major contribution to immediate defenses, which are critical for a species like rice that can only endure transient exposure to milder cold stress.

Second, our current data revealed a number of oxidative-mediated early response expression clusters associated with specific classes of regulatory sequences such as as1/ocs/TGA-like, GCC-box/JAre-like and Myb2-box-like cis-elements. Several classes of TFs are likely to act on these clusters including bZIP Group-D (TGA), Group-S (ocs), and Group-I (GA-associated), ERF Group-I (subfamily A-6), Group-IV (subfamily A-2), Group-VI (subfamily B-5), Group-VII (subfamily B-2), Group-X (subfamily B-4), and R2R3-MYB [24,29,37,60]. Known interactions of these TFs with such elements and their involvement in various oxidative mediated processes justify their presumed roles as regulators of early response transcriptome. Particularly interesting candidates for regulon engineering are the as1/ocs/TGA-like element-regulated clusters comprising the largest group of genes associated with oxidative defenses.

Third, we have identified regulatory clusters that appear to be independent of oxidative signals. Two of the well known regulons that function during cold acclimation at 4°C in *Arabidopsis *were among those and they are the ABRE-like (bZIP-ABF) and CRT/DRE/rav1-like (*CBF/DREB/RAP2/RAV1*) element-enriched clusters. The third cluster (GARE/pyrimidine-box-like element-enriched) which involves an R1-MYB appears to be a component of combinatorial control mechanism. ABRE-enriched clusters appear to be downstream to the oxidative-mediated clusters and this is consistent with the timing of ABA response, i.e., after 24 hours. Recent report estimated that 400 protein-coding genes of rice are regulated via an ABRE requiring mechanism [103]. This number is close to the estimated number of genes that belong to the chilling induced ABRE-enriched clusters, which is much less than the estimated number of genes regulated by an oxidative mediated mechanism. This data provide additional support that oxidative-mediated mechanism is the primary route for activating early response genes.

The cold acclimation associated regulons controlled by *DREB/CBF, RAP2 *and *RAV1 *are induced maximally at 4°C in *Arabidopsis *[13,42,43]. Apparently, orthologous TFs in rice are induced at 10°C, which probably induce a different set of target genes distinct from those that are activated in cold acclimating species. An interesting trend that we observed was that chilling upregulated clusters in rice have only moderate enrichment of DRE/CRT/rav1-like elements compared to other types of elements. The DRE/CRT/rav1-like elements associated clusters are also enriched with one or more other types of elements. A possible explanation is that at milder cold stress, *DREB/CBF/RAP2/RAV1-*regulated genes account for a relatively smaller fraction of the transcriptome compared to the oxidative mediated components. It has also been suggested that the genomes of chilling insensitive (e.g., *Arabidopsis*) and sensitive (e.g., rice) species might differ in terms of the distribution of DRE/CRT/rav1-like elements [42].

Fourth, our data showed an important role of combinatorial control in chilling stress response gene expression. Combinatorial control has long been recognized and numerous examples are constantly being reported in relation to stress response [17,26,77,104]. Interaction of several classes of TFs facilitates fine-tuned regulation. Examples of these are the possible roles of WRKY TFs as repressors of ABA-mediated gene expression, which allows fine-tuned regulation of oxidative mediated transcriptome in relation to ABA-mediated responses. Interaction of several TF classes also facilitates integration of various signals associated with stress, growth and development. GARE/pyrimidine-box-like elements associated with R1-MYB TFs were the most widely distributed elements among the chilling upregulated clusters. This class of elements occurred in various combinations with other classes. R1-MYB TFs are primarily involved in gibberellic acid regulated growth processes [79,82,83]. R1-MYB factors appear to coordinate and fine-tune gene expression required for growth modulation and survival at sub-optimal temperature, which appear to be a key aspect of vigor enhancement and/or maintenance under stress conditions.

Finally, our results provide a global picture of biochemical and physiological consequences of the regulatory networks induced at 10°C. The obvious theme is the striking similarities of chilling-induced processes to those associated with defenses against pathogens and responses to wounding. The predominance of genes involved in disease response signaling was indicated by large number of LRR, NBS-LRR, NB-ARC types of receptor proteins, genes associated with hypersensitive response, wounding, herbivory, redox regulation, cellular detoxification, programmed cell death, and terpenoid metabolism most of which are linked to jasmonic acid, salicylic acid and ethylene signaling. Activities of these genes provide further evidence that oxidative signal was the origin of most of the early responses.

The agronomic significance of the current results is quite interesting. The data provide a global picture of how stress, growth and developmental responses are integrated by the interaction of at least four major classes of TF. The interesting relationship between TF expression and QTL for stress tolerance and seedling vigor might be the key for further understanding of the precise mechanisms that make japonica rice withstand chilling better than indica rice. Genes related to stress response, growth, development and energy partitioning are often enriched within the boundaries of QTL associated with vigor and yield. An emerging theme is that linkage blocks with certain allelic combinations of stress-related genes involved in responses to diseases, dehydration, temperature extremes, and light stress are important components of plant vigor and heterosis [18,105-108]. Modulated growth, physiological sustenance and maintenance of vigor appear to be the downstream consequences of the transcriptional networks induced by chilling, providing a means to delay the occurrence of irreversible injuries. This hypothesis is consistent with the fact that many japonica cultivars are able to survive chilling for a much longer period of exposure than most indica cultivars.

## Methods

### Plant materials, growth conditions, and stress treatments

Rice cultivars with contrasting sensitivities to chilling, i.e., Nipponbare and CT6748 (less sensitive) and INIAP12 and IR36 (more sensitive) were used in this study. Seedlings were grown to three-leaf (V_3_) stage at optimum temperature (28°C) before exposure to chilling condition (10°C) in a Pervical E30BHO growth chamber (Percival Scientific, Perry, IA). These experiments were performed based on previously described methods [14,17]. For the H_2_O_2 _experiment, seedlings were grown to V_3 _stage in standard Yoshida hydroponic solution at 28°C. Experimental plants were transferred to fresh medium with 4 mM H_2_O_2 _while the control plants were maintained in medium without H_2_O_2_. Tissues were harvested after 1, 3 and 6 hours of treatment and another 6 hours of recovery in fresh medium without H_2_O_2_. Total RNA was isolated with Trizol reagent (Invitrogen, Carlsbad, CA) from leaves after 0.5, 2, 4, 6, 12, 18, 24, 36, 48 and 96 hours of exposure to chilling and after each sampling time in the H_2_O_2 _experiment. Seedling survival analysis was performed as previously described [14].

### Microarray analysis

RNA samples from control and experimental Nipponbare seedlings were processed with the MessageAmp II aRNA amplification and T7 polymerase *in vitro *transcription kits producing at least 20 μg of aminoallyl-dUTP-labeled aRNA. The cDNA samples were synthesized with oligo-dT primer and ArrayScript reverse transcriptase. All procedures were according to manufacturer's instructions (Ambion, Austin, TX). Equal amounts of Cy5- and Cy3-labeled samples were combined in 1× hybridization buffer composed of 50% formamide, 5× SSC, 0.1% SDS and 10 mM DTT. Chemical labeling with Cy3 (control) and Cy5 (treatment) dyes was performed according to manufacturer's protocol (GE Healthcare-Amersham, Piscataway, NJ).

The NSF rice oligonucleotide microarray version 3, which contains roughly 45,000 probes representing all 40,000 predicted genes of japonica rice was used in all experiments. Pre-hybridization treatments were according to manufacturer's instructions [19]. Hybridization was performed for 18 hours at 42°C in Hybex Humidified Thermal Blocks (Scigene, Sunnyvale, CA). Stringency washing was performed at 42°C in 2× SSC, 0.1% SDS and 0.1× SSC, 0.1% SDS and at room temperature in 0.1× SSC for 10 minutes each step. Expression data was acquired with the Axon 4000A scanner and processed with the GenePix Pro 5.1 (Axon-Molecular Devices, Sunnyvale, CA). Two independent biological replicates were performed for each control by treatment comparison. Dye-swap experiments were initially performed to assess dye by sample interaction, which was found to be negligible. Gene expression data was normalized globally prior to high level analysis. Microarray dataset can be accessed by GSE8767 and GSE10062 at the Gene Expression Omnibus [109]. Values represent the log_2 _ratio of background subtracted intensity values (Cy5/Cy3). High level statistical analyses (T-test, hierarchical and K-mean clustering) were performed with the MeV Bioinformatics Tools [110]. Protein-coding genes with log_2 _fold ≥ 1.8 (p < 0.05) in at least two consecutive time points for the chilling stress experiment and at least one time point for the H_2_O_2 _experiment were first identified from the total dataset. Genes that passed these criteria comprised the total gene set for high-level analysis. TFs were identified and grouped according to family according to the Database of Rice Transcription Factors [22]. Members of each family were hierarchically clustered while the NTFs were divided into smaller groups by K-mean clustering. NTFs were further classified by functional categories using the RiceCyc pathway [21] and Interpro protein domain [87] databases. Co-location and enrichment of candidate genes within the boundaries of relevant QTL was performed with Fisher exact test based on current information in Gramene QTL database [21].

### Ab initio promoter analysis

Promoter motif enrichment analysis was performed on the entire set of upregulated NTFs (2,456) and individually for each K-mean cluster [17]. Sequences of *bona fide *promoter regions (-1,000 to +200) were extracted from the Nipponbare genome sequence by locating the experimentally validated transcription start site (TSS) by alignment with FLcDNA [111]. The Dragon Motif Builder algorithm with EM2 option was used to detect over-represented motifs [112]. Thirty motifs (8 to 10 nt) were detected each run with a threshold value of 0.875. Promoters of randomly selected genes not associated with stress response mechanisms were used for background subtraction of random motif occurrence. Significant motifs were selected based on a threshold occurrence of 50%. Motif classes were identified by significant matches with TRANSFAC [113,114], PLACE [115] and AGRIS [116,117] databases.

### Real-time PCR analysis

Comparative analysis of selected chilling upregulated genes between Nipponbare and INIAP12 was performed by real-time PCR as previously described [17]. PCR reactions were performed with the Verso cDNA synthesis and Absolute QPCR Sybr Green mix (AbGene, Rochester, NY) using the myCycler real-time PCR system (Biorad, Hercules, CA).

### H_2_O_2 _concentration assay

Total H_2_O_2 _content of rice leaves was determined by the Amplex Red (10-acetyl-3,7-dihydroxyphenoxazine) assay (Invitrogen, Carlsbad, CA) according to established procedures [18]. Leaf discs were homogenized in chilled 5% TCA. Crude extracts were centrifuged at 12,000×g for 10 minutes and further purified with Dowex anion exchange resin (AG1X100). Samples were buffered to pH 7.4 with 0.25M NaH_2_PO_4_. Concentration was measured every 30 minutes by the absorbance at 560 nm with three replicates.

### Electrophoretic mobility shift assay (EMSA)

Nuclear protein extraction was performed based on established procedures [118,119]. Proteins were extracted from leaf tissues (10 g) after exposure to chilling (10°C) for 6, 12 and 24 hours and quantified by Bradford method. EMSA was performed with the Gel Shift Assay System (Promega, Madison, WI) following the manufacturer's protocol. Double stranded oligonucleotide probes were synthesized and end-labeled with [γ-^32^P]-dATP (3,000Ci/mmol) by T4 polynucleotide kinase. Unlabelled probes were removed using the Bio-Spin 6 (Bio-Rad, Hercules, CA). Binding reactions (10 μg nuclear extract +1 μl ^32^P-labeled probe) were carried out for 30 minutes at room temperature. For competition experiments, excess unlabeled probes were added in approximately eight-fold molar ratio relative to labeled probes. Binding reactions were analyzed by electrophoresis in a 4% non-denaturing polyacrylamide gel.

## Authors' contributions

KYY and MRP performed the microarray experiments including all statistical and bioinformatic analyses. RM and RB performed the gene ontology (GO) and QTL enrichment analyses. BM, EW and VBB performed the *ab initio *promoter analysis. VH contributed to the microarray and RT-PCR analyses and performed the leaf H_2_O_2 _quantitation experiments. FX performed the genotypic comparisons by RT-PCR and EMSA experiments. BGDR was responsible for the overall concept and experimental designs, data integration, analysis and interpretation, and manuscript preparation. All authors approved the final manuscript.

## Supplementary Material

Additional file 1 **Dominant functional categories in the downregulated group of genes**. This data shows the most highly enriched broad functional categories of downregulated genes classified according to gene ontology. Click here for file

Additional file 2 **Expression matrix of chilling upregulated WRKY transcription factors**. Heat map showing the temporal expression profiles of WRKY transcription factors under chilling stress. Gene designations were based on putative *Arabidopsis *orthologs according to the most recent genome annotation. Click here for file

Additional file 3 **Expression matrix of chilling upregulated bHLH transcription factors**. Heat map showing the temporal expression profiles of bHLH transcription factors under chilling stress. Gene designations were based on putative *Arabidopsis *orthologs according to the most recent genome annotation. Click here for file

Additional file 4 **Expression matrix of chilling upregulated NAC transcription factors**. Heat map showing the temporal expression profiles of NAC transcription factors under chilling stress. Gene designations were based on putative *Arabidopsis *orthologs according to the most recent genome annotation. Click here for file

Additional file 5 **Functional categories and grouping by cluster of chilling upregulated NTF genes**. List of all NTF genes included in the analysis of the chilling upregulated transcriptome grouped according to putative functions. Click here for file

Additional file 6 **Correlation between timing of induction and function of chilling responsive genes**. Distribution of functional categories in relation to activation timing of co-expressed gene clusters. (A) Signaling and response regulation; (B) cellular defense and rescue; (C) physiological adjustment and sustenance mechanisms. Gray: rapid phase-1 clusters; Red: phase-2 and phase-3 clusters. Click here for file

Additional file 7 **Relative enrichment of other types of cis-elements detected in the promoters of chilling upregulated genes**. List of cis-elements associated with other types of transcription factors not included in Tables 2, 3 and 4. Click here for file

Additional file 8 **Components of the chilling stress transcriptome with possible roles in oxidative stress and redox regulation**. List of upregulated genes with possible roles in oxidative stress and redox regulation classified according to gene ontology. Click here for file

Additional file 9 **Components of the chilling stress transcriptome with possible roles in response to diseases, elicitors and apoptosis**. List of upregulated genes with possible roles in response to diseases, elicitors and apoptosis classified according to gene ontology. Click here for file

Additional file 10 **Components of the chilling stress transcriptome with possible roles in cellular detoxification and efflux**. List of upregulated genes with possible roles in cellular detoxification and efflux classified according to gene ontology. Click here for file

Additional file 11 **Possible components of physiological adjustment and sustenance mechanisms**. Functional categories relevant to physiological adjustment and sustenance processes classified according to gene ontology. Click here for file

Additional file 12 **Association between chilling-induced transcription factors and stress-associated QTL**. Genomic location of chilling upregulated transcription factors relative to the boundaries of known QTL of rice associated with seedling vigor and stress response. Click here for file
