# Supplementary figures and images for: Transcriptional regulatory network triggered by oxidative signals configures the early response mechanisms of japonica rice to chilling stress
Source: BMC Plant Biol. 2010 Jan 25;10:16. doi: 10.1186/1471-2229-10-16 (PMC2826336; doi:10.1186/1471-2229-10-16)

## Slide 1
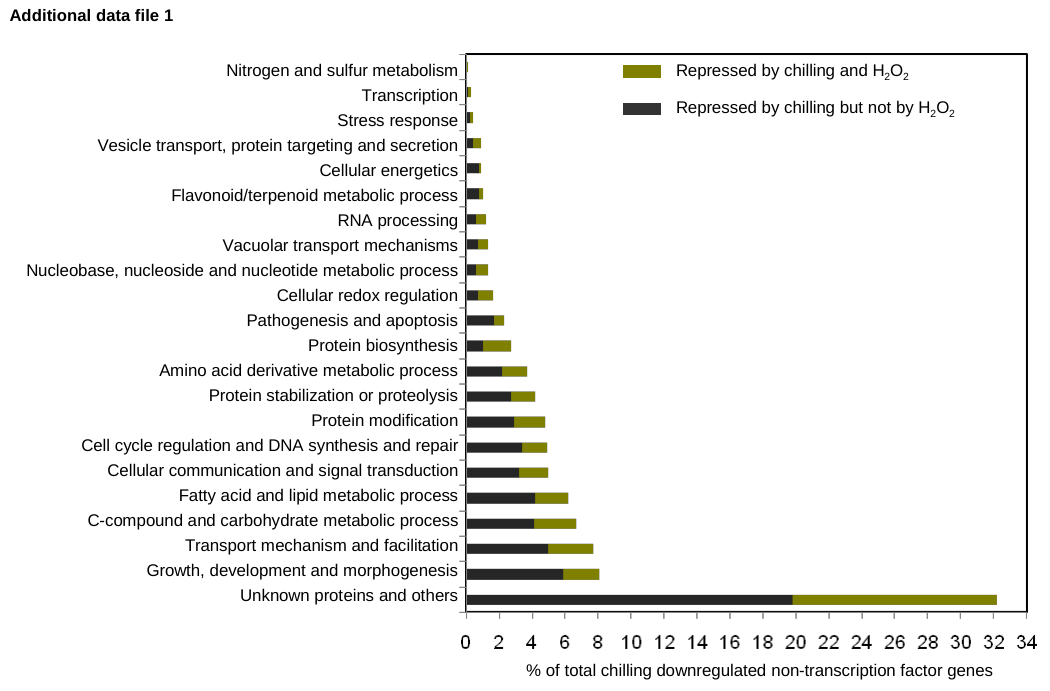

Additional data file 1

Supplement: Additional file 1 — Dominant functional categories in the downregulated group of genes. This data shows the most highly enriched broad functional categories of downregulated genes classified according to gene ontology. [file 1471-2229-10-16-S1.PPT]

## Slide 1
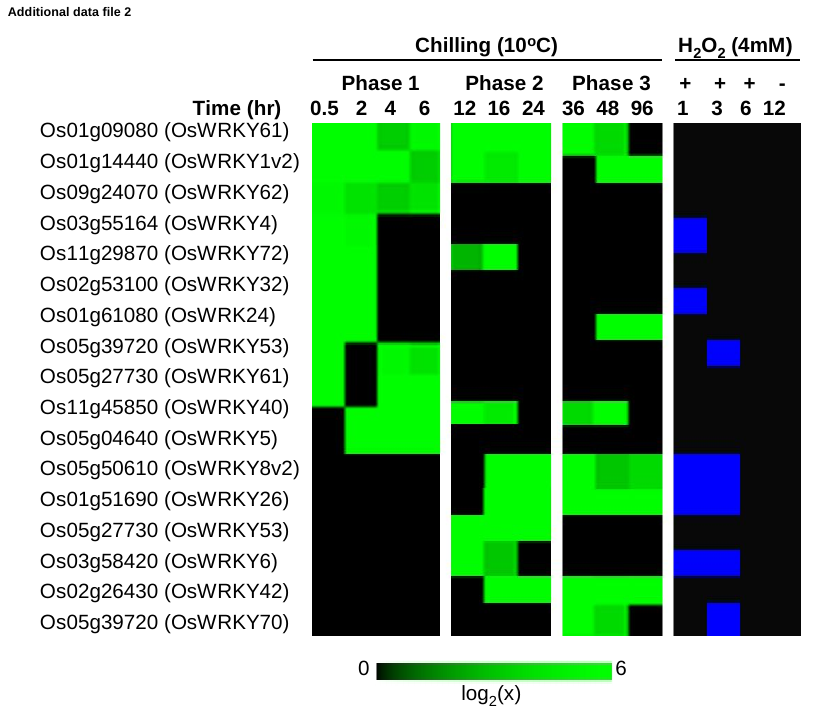

Additional data file 2

Supplement: Additional file 2 — Expression matrix of chilling upregulated WRKY transcription factors. Heat map showing the temporal expression profiles of WRKY transcription factors under chilling stress. Gene designations were based on putative Arabidopsis orthologs according to the most recent genome annotation. [file 1471-2229-10-16-S2.PPT]

## Slide 1
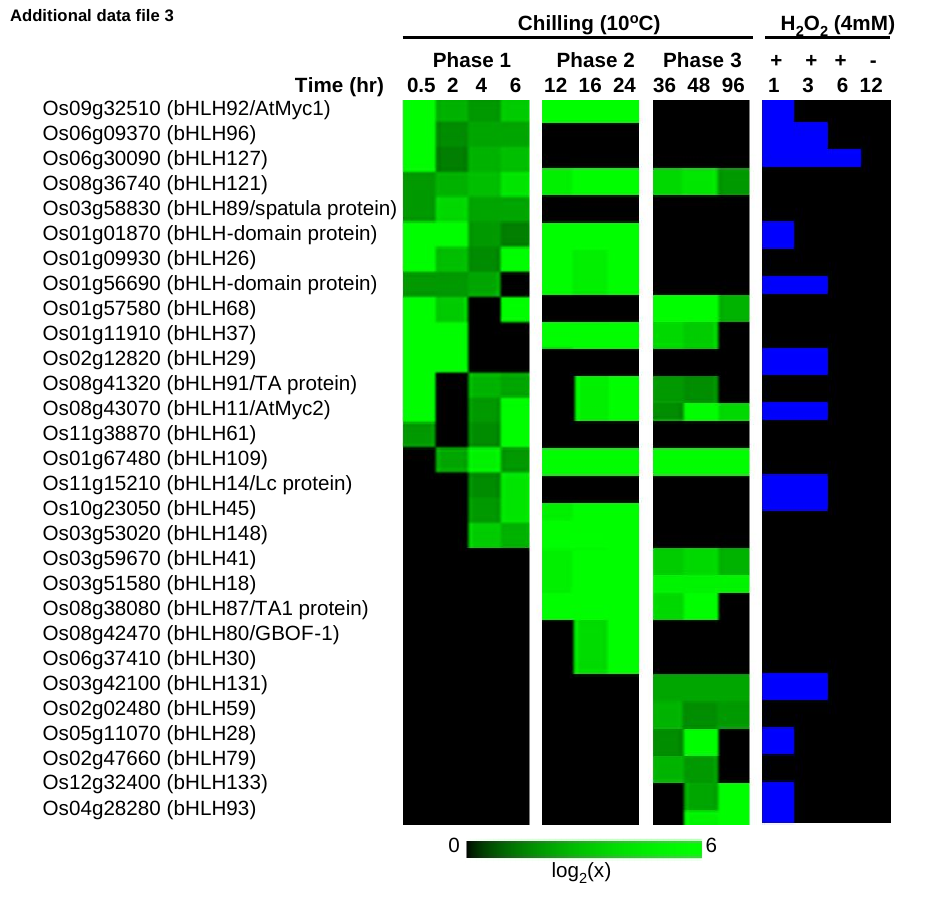

Additional data file 3

Supplement: Additional file 3 — Expression matrix of chilling upregulated bHLH transcription factors. Heat map showing the temporal expression profiles of bHLH transcription factors under chilling stress. Gene designations were based on putative Arabidopsis orthologs according to the most recent genome annotation. [file 1471-2229-10-16-S3.PPT]

## Slide 1
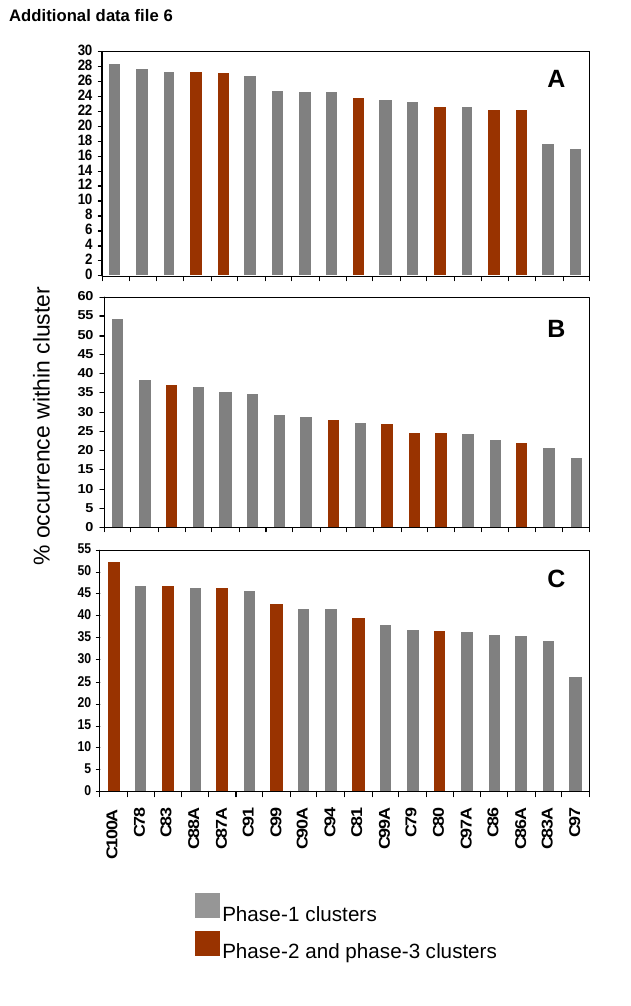

Additional data file 6

Supplement: Additional file 6 — Correlation between timing of induction and function of chilling responsive genes. Distribution of functional categories in relation to activation timing of co-expressed gene clusters. (A) Signaling and response regulation; (B) cellular defense and rescue; (C) physiological adjustment and sustenance mechanisms. Gray: rapid phase-1 clusters; Red: phase-2 and phase-3 clusters. [file 1471-2229-10-16-S6.PPT]

## Slide 1
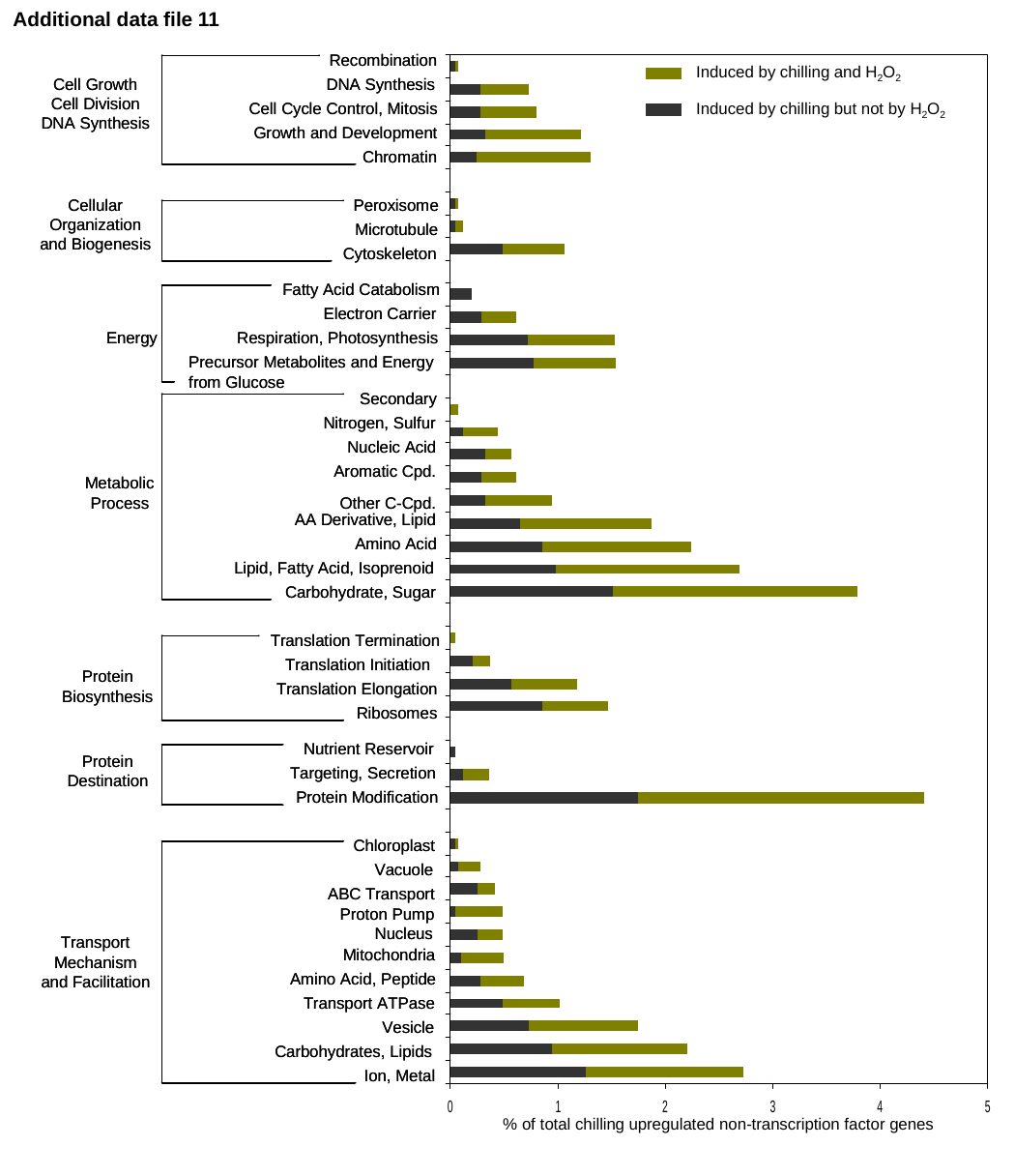

Additional data file 11

Supplement: Additional file 11 — Possible components of physiological adjustment and sustenance mechanisms. Functional categories relevant to physiological adjustment and sustenance processes classified according to gene ontology. [file 1471-2229-10-16-S11.PPT]
