# Supplementary material for: Transcriptional regulatory network triggered by oxidative signals configures the early response mechanisms of japonica rice to chilling stress
Source: BMC Plant Biol. 2010 Jan 25;10:16. doi: 10.1186/1471-2229-10-16 (PMC2826336; doi:10.1186/1471-2229-10-16)
Supplement: Additional file 4 — Expression matrix of chilling upregulated NAC transcription factors. Heat map showing the temporal expression profiles of NAC transcription factors under chilling stress. Gene designations were based on putative Arabidopsis orthologs according to the most recent genome annotation. [file 1471-2229-10-16-S4.PPT]

## Slide 1
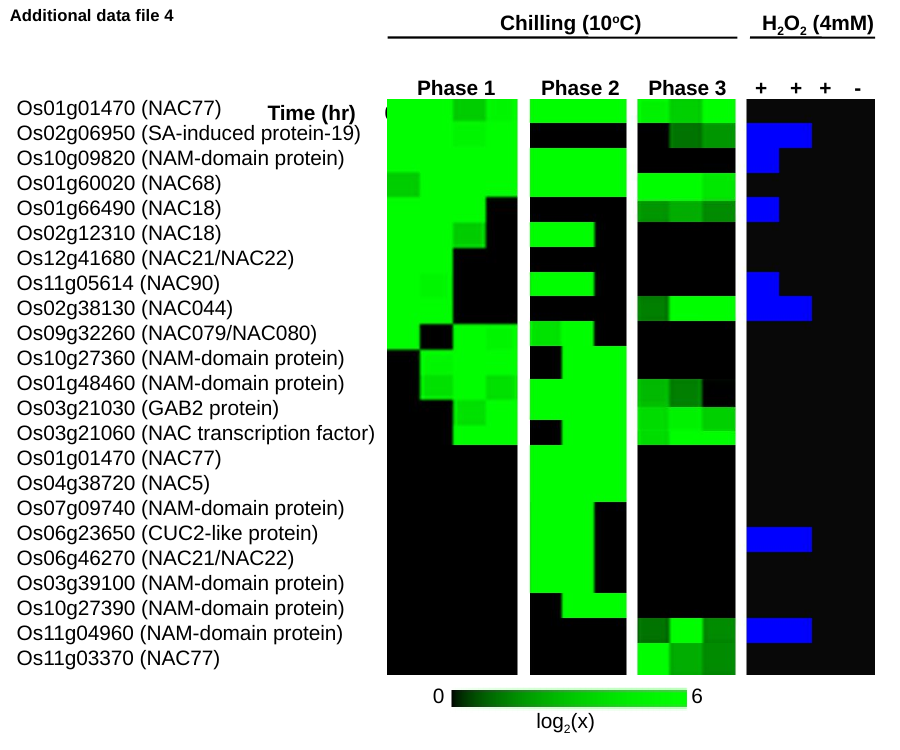

Additional data file 4
 Chilling (10oC) H2O2 (4mM)
 Phase 1 Phase 2 Phase 3 + + + -
 Time (hr) 0.5 2 4 6 12 16 24 36 48 96 1 3 6 12
Os01g01470 (NAC77)
Os02g06950 (SA-induced protein-19)
Os10g09820 (NAM-domain protein)
Os01g60020 (NAC68)
Os01g66490 (NAC18)
Os02g12310 (NAC18)
Os12g41680 (NAC21/NAC22)
Os11g05614 (NAC90)
Os02g38130 (NAC044)
Os09g32260 (NAC079/NAC080)
Os10g27360 (NAM-domain protein)
Os01g48460 (NAM-domain protein)
Os03g21030 (GAB2 protein)
Os03g21060 (NAC transcription factor)
Os01g01470 (NAC77)
Os04g38720 (NAC5)
Os07g09740 (NAM-domain protein)
Os06g23650 (CUC2-like protein)
Os06g46270 (NAC21/NAC22)
Os03g39100 (NAM-domain protein)
Os10g27390 (NAM-domain protein)
Os11g04960 (NAM-domain protein)
Os11g03370 (NAC77)
 0 6
 log2(x)
