# Supplementary material for: Transcriptional regulatory network triggered by oxidative signals configures the early response mechanisms of japonica rice to chilling stress
Source: BMC Plant Biol. 2010 Jan 25;10:16. doi: 10.1186/1471-2229-10-16 (PMC2826336; doi:10.1186/1471-2229-10-16)
Supplement: Additional file 7 — Relative enrichment of other types of cis-elements detected in the promoters of chilling upregulated genes. List of cis-elements associated with other types of transcription factors not included in Tables 2, 3 and 4. [file 1471-2229-10-16-S7.PDF]

Additional file 7. Relative enrichment of other types of cis-elements in promoters.

Cluster	Motif	Putative element[a]	Associated class of bZIP (putative)[b]	% (TIC)[c]	e-value
C100A	AAAATTTG	Unknown	Unknown	70 (12.98)	2e-004
	TGCTACTC	MYC2-like	bHLH (JA-MYC)	50 (12.14)	1e-003
	ATGAGAGAG	MYC2-like	bHLH (JA-MYC)	53 (12.89)	2e-004
C99	ATGGTTCA	SIF box-like	SIF	73 (10.83)	5e-004
	AAAAGGGA	TAAG element-like	Dof1	68 (12.52)	5e-004
	GAGTGGAT	MADS box-like	MADS	64 (11.90)	3e-004
	CCCCCCTC	Unknown	Unknown	62 (13.23)	6e-005
	CGTCGTCG	Unknown	Unknown	57 (13.80)	3e-005
C99A	AAAAAATG	PF1 element-like	PF1	66 (12.99)	5e-004
	GAGGAAGG	Unknown	Unknown	64 (12.78)	1e-004
	GCCATGCT	AuxRE-like	ARF	62 (11.13)	8e-005
C97	(a/c)TCCCATC	PE3 element-like	Unknown	59 (11.89)	8e-004
	TCCATGT(a/g)	ERD element-like	NAC	50 (12.23)	3e-004
	GGGT(g/t)(A_4)	W-box-like	WRKY	50 (12.82)	3e-004
C97A	TAATATC	Unknown	Unknown	64 (10.94)	5e-004
	CTCCTCCT	Unknown	Unknown	62 (14.04)	8e-004
	CATGTGAT	ERD element-like	NAC	52 (12.35)	2e-004
C94	CAACCCCA	CAACC-box-like	bHLH (MYC)	71 (11.52)	5e-004
	AGGGGAGG	Unknown	Unknown	50 (12.22)	1e-004
C91	CACCCCCA	ACII element-like	Unknown	76 (11.23	4e-004
	TTCATGTG	ERD element-like	NAC	66 (12.17)	6e-005
	AGAGATAAA	GATA element-like	GATA	61 (13.60)	1e-004
	AGAGCTTT	Unknown	Unknown	55 (11.64)	3e-003
	AGAAATTG	W-box-like	WRKY	53 (12.49)	2e-004
C90A	GAACGATG	Unknown	Unknown	67 (11.18)	2e-003
	AGGAATTG	W-box-like	WRKY	65 (11.51)	2e-004
	GGTTTGAC	W-box-like	WRKY	56 (11.47)	1e-004
	AATTACTCC	MYC2-like	bHLH (MYC)	56 (12.27)	3e-004
	AAAATTAG	Vspb-like	HD-ZIP	52 (12.62)	2e-004
C88A	AGGAAGAA	GATA element-like	GATA	55 (13.38)	2e-004
	AAGAGGAG	Unknown	Unknown	55 (13.46)	3e-004
	TGCTACTG	Unknown	Unknown	54 (12.17)	1e-004
	CCCTCTCCTC	Unknown	Unknown	50 (15.46)	4e-004
C88A	AGGAAGAA	GATA element-like	GATA	55 (13.38	2e-004
	AATAAAAT	Unknown	Unknown	53 (12.81)	2e-004
	CCCTCTCCTC	Unknown	Unknown	50 (15.46)	4e-004
C87A	TTTGTAAA	AuxRE-like	ARF	71 (12.42)	8e-004
	GCCATGATC	AuxRE-like	ARF	58 (11.75)	4e-004
	CAGCAGCA	Unknown	Unknown	58 (12.86)	3e-004
C86	TTCATCAA	CArG-box-like	MADS	80 (11.37)	1e-003
	TAATTGCA	W-box-like	WRKY	59 (11.93)	5e-004
C86A	CAAAAAAA	Unknown	Unknown	58 (13.90)	2e-004
	TATACTCC	MYC2-like	bHLH (MYC)	50 (13.51)	2e-004
C83	AAATAAA	AuxRE-like	ARF	70 (13.48)	1e-003
	TA(a/c/g)AT	Unknown	Unknown	67 (10.80)	7e-004
	TTG(a/t)(a/g/t)GAT	Unknown	Unknown	64 (11.64)	4e-004
	G(c/t)CAAAT(c/g)G	W-box-like	WRKY	50 (11.95)	7e-004

Cluster	Motif	Putative element[a]	Associated class of bZIP (putative)[b]	% (TIC)[c]	e-value
C80	GAAGAAGT	GATA element-like	GATA	72 (11.43)	3e-004
	AGGAGGAG	Unknown	Unknown	61 (13.79)	3e-005
	CTCCCTCT	Unknown	Unknown	57 (13.31)	5e-004
	GGAGGGAG	Unknown	Unknown	52 (13.78)	3e-004
C79	TTCCTGCTC	Unknown	Unknown	59 (12.42)	6e-004
	TTTAGGCT	Unknown	Unknown	54 (12.39)	2e-004
	TGATCTTG	Unknown	Unknown	54 (12.25)	3e-004
	TAGTAGGA	Unknown	Unknown	54 (11.84)	1e-003
C78	AAAAGGGA	TAAG element-like	Dof1	68 (12.52)	5e-004
	GGGTGGAT	MADS box-like	MADS	64 (11.90)	3e-004
	CCCCCCTC	Unknown	Unknown	62 (13.23)	6e-005
	CGTCGTCG	Unknown	Unknown	57 (13.60)	3e-005

[a]Based on homology with motifs that have been identified in other plant species (TRANSFAC, PLACE and AGRIS databases).
[b]Possible classes of associated transcription factors based on database information.
[c]Percentage occurrence in critical promoters relative to background sequences (Total Information Content).
