# Supplementary material for: Transcriptional regulatory network triggered by oxidative signals configures the early response mechanisms of japonica rice to chilling stress
Source: BMC Plant Biol. 2010 Jan 25;10:16. doi: 10.1186/1471-2229-10-16 (PMC2826336; doi:10.1186/1471-2229-10-16)
Supplement: Additional file 8 — Components of the chilling stress transcriptome with possible roles in oxidative stress and redox regulation. List of upregulated genes with possible roles in oxidative stress and redox regulation classified according to gene ontology. [file 1471-2229-10-16-S8.PDF]

Additional file 8. Genes associated with oxidative stress and redox regulation based on relevant Gene Ontology (GO) and Interpro (IPRO) terminologies.

**Annotation**	**GO/IPRO terminology**	**Induced by chilling but not H_2O_2**	**Induced by chilling and H_2O_2**
2-cys peroxiredoxin (chloroplast)	Peroxidase/antioxidant activities (GO_0016209; GO_0004601)	Os02g33450	
Acetyl-coA carboxylase ACC1A	Response to oxidative stress (GO_0006979); Peroxidase activity (GO_0004601)	Os10g21910	
AhpC/TSA protein	Antioxidant activity (GO_0016209)	Os01g48420	
L-ascorbate proxidase-9	Peroxidase activity (GO_0004601); Response to oxidative and abiotic stress (GO_0006979, GO_0009607)	Os12g07820	
Cationic peroxidase-1	Peroxidase activity (GO_0004601); Response to oxidative and abiotic stress (GO_0006979, GO_0009607)	Os02g14440	Os11g02100
Dishevelled, Egl10, and Pleckstrin	Cell redox homeostasis (GO_0045454)	Os10g34170	
Ferric reductase-like protein	IPRO_Respiratory burst flavocytochrome protein	Os04g36720	
Ferritin-1	Oxidoreductase activity (GO_0016491): Response to stress (GO_0006950);		Os12g01530
Flavodoxin	Antioxidant activity (GO_0016209)		Os03g53730
Glutaredoxin	Cell redox homeostasis (GO_0045454)	Os07g46570<br>Os02g40500<br>Os04g42930<br>Os08g45140	
Glutaredoxin-like protein	Cell redox homeostasis (GO_0045454)	Os12g35340	
Glutathione peroxidase	Peroxidase activity (GO_0004601); Response to oxidative and abiotic stress (GO_0006979, GO_0009628)	Os03g24380	
Glutathione S-transferase	Glutathione peroxidase activity (GO_004602); Response to oxidative and abiotic stress (GO_0006979, GO_0009628)	Os08g43680<br>Os05g34150<br>Os07g07320<br>Os10g34020	Os10g38780<br>Os09g37240<br>Os01g49710<br>Os01g72120<br>Os10g25590<br>Os10g22070
Hsc70 interacting protein	Cell redox homeostasis (GO_0045454)		Os09g23650
Lactoglutatione lyase	IPRO_ROS scavenger	Os07g06660	
Metallothionein-like protein type 2	Response to stress (GO_0006950); Metal ion binding (GO_0046872)		Os01g05650
Multicopper oxidase	Response to stress (GO_0006950); Metal ion binding (GO_0046872)	Os10g30140<br>Os01g03630	
NADP dependent oxidoreductase P1	Response to stress (GO_0006950)		Os12g12470
NADPH oxidase	Response to stress (GO_0006950)		Os01g53294

Annotation	GO/IPRO terminology	Induced by chilling but not H_2O_2	Induced by chilling and H_2O_2
NADPH:quinone oxidoreductase	IPRO_Oxidative cycling with superoxide generation	Os01g72460	
Adenosine 5'-phosphosulfate reductase	Cell redox homeostasis (GO_0045454)		Os03g59170
Peroxidase	Peroxidase activity (GO_0004601); Response to oxidative and biotic stress (GO_0006979, GO_009607)	Os01g07770<br>Os11g10460<br>Os06g13050<br>Os08g41090<br>Os01g02070	Os06g48020<br>Os03g55410<br>Os05g04410<br>Os05g04500<br>Os05g14260<br>AK120047<br>AK064633
Phospholipid hydroperoxide glutathione peroxidase	Glutathione peroxidase activity (GO_004602); Response to oxidative and abiotic stress (GO_0006979, GO_0009628)		Os04g46960
Respiratory burst oxidase	IPRO_Free radical reductase; Oxidoreductase activity (GO_0016491)		Os01g61880
Senescence protein	IPRO_senescence related	Os07g17330	
Squalene monoxygenase	Oxidoreductase activity (GO_0016491); Response to stress (GO_0006950)	Os03g12910	
Superoxide dismutase	Superoxide metabolism (GO_0006801); Response to stress (GO_0006950)	Os06g02500	Os08g44770
Thioredoxin family protein	Cell redox homeostasis (GO_0045454)	Os03g55820<br>Os09g38670<br>Os01g68480<br>Os04g44830<br>Os03g21000	Os06g45510<br>Os05g11990<br>Os05g07690
