# Supplementary material for: Transcriptional regulatory network triggered by oxidative signals configures the early response mechanisms of japonica rice to chilling stress
Source: BMC Plant Biol. 2010 Jan 25;10:16. doi: 10.1186/1471-2229-10-16 (PMC2826336; doi:10.1186/1471-2229-10-16)
Supplement: Additional file 9 — Components of the chilling stress transcriptome with possible roles in response to diseases, elicitors and apoptosis. List of upregulated genes with possible roles in response to diseases, elicitors and apoptosis classified according to gene ontology. [file 1471-2229-10-16-S9.PDF]

Additional file 9. Genes associated with disease, elicitor, and wounding responses based on relevant Gene Ontology (GO) and Interpro (IPRO) terminologies.

**Annotation**	**GO/IPRO terminology**	**Induced by chilling but not H_2O_2**	**Induced by chilling and H_2O_2**
Acyl activating enzyme 11	IPRO_JA biosynthesis; Response to biotic and abiotic stimuli (GO_0009607, GO_0009628)		Os03g03790
ATPase AAA-2	Response to stress (GO_0006950); Apoptosis (GO_0006915)	Os03g05730	
Autophagy protein	IPRO_Apoptosis; Autophagic vacuole (GO_0000045)		Os06g10340<br>Os03g14380
Avr9 elicitor protein	IPRO_Response to elicitors	Os09g27950	
BAG domain protein	Apoptosis (GO_0006915)		Os11g31060
B-cell receptor 31	Apoptosis (GO_0006915)	Os05g19040	
Benzoate carboxyl methyltransferase	Response to biotic stress (GO_0006950, GO_0009607)	Os06g13560	
Beta-1,3-glucanase	IPRO_pathogenesis related		Os01g71930
Caffeoyl-CoA O-methyltransferase	IPRO_Phenylpropanoid biosynthesis	Os06g06980	Os08g38900
Chalcone/stilbene synthase	IPRO_Phytoalexin synthesis		Os07g17010
Chalcone isomerase	IPRO: Flavonoid biosynthesis	Os02g21520	
Chitinase	Pathogenesis related (GO_0006032)		Os10g28050<br>Os08g41100
CPR5 protein	Response to biotic stimulus (GO:0009607); Cell death (GO:0008219); IPRO_Apoptosis	Os01g68970	
Dihydroflavonol-4-reductase	IPRO_Detoxification and cell death resistance	Os09g09270	
Dirigent-like disease resistance protein RGA3	IPRO_Disease resistance; Defense to fungi and bacteria (GO_0042742, GO_0050832)	Os02g16100	Os01g25030
Endochitinase 26kDa	IPRO_Pathogenesis related; Cell death (GO_0008219)	Os01g18400	
Cupin	IPRO_Pathogenesis related	Os04g36760	
Epsin	IPRO_Endocytosis	Os08g45230	
Elicitor response protein	Response to stress (GO_0006950, GO_0009628)	Os04g44870	Os01g62430
Flavonoid monooxygenase	Response to abiotic stimuli (GO_0009628, GO:0009719)		Os10g16974
Flavonol sulfotransferase	IPRO_Biosynthesis of heparan sulfates and heparin		Os08g40390
Germin-like protein	Response to abiotic stress (GO_0006950, GO_0009628)	Os04g52720<br>Os11g33100	Os08g13440
Harpin binding protein	IPRO_Pathogenesis related		Os11g38260
Harpin induced protein	IPRO_Pathogenesis related		Os01g64470<br>Os02g33550<br>Os02g16610
HCBT defense protein	IPRO_Pathogenesis related	Os08g02030	

**Annotation**	**GO/IPRO terminology**	**Induced by chilling but not H_2O_2**	**Induced by chilling and H_2O_2**
Hemolysin A	Hemolysis (GO_0019836)	Os11g14230	
Hevamine A	Chitin catabolism (GO_0006032)		Os01g64110
Integral membrane protein	Pathogenesis related (GO_0009405);<br>Response to biotic stimulus (GO_0006810)	Os10g33920	Os02g41780
LRR family protein	Kinase activity (GO_0016301);<br>Response to biotic and abiotic stimuli (GO_0009607, GO_0009628);<br>Response to endogenous stimuli (GO_0009719)	Os01g06890<br>Os01g02060<br>Os01g06790<br>Os01g68870<br>Os02g06150<br>Os02g17730<br>Os02g05930<br>Os02g05970<br>Os02g13510<br>Os02g13640<br>Os03g40250<br>Os03g54900<br>Os04g08370<br>Os04g03180<br>Os04g15600<br>Os06g08710<br>Os06g38990<br>Os07g03000<br>Os08g34640<br>Os08g39570<br>Os10g25487<br>Os10g38450<br>Os10g42190<br>Os10g22930<br>Os11g42660	Os01g06720<br>Os01g06820<br>Os01g33430<br>Os01g05980<br>Os01g04720<br>Os02g13460<br>Os02g05980<br>Os03g12250<br>Os03g03570<br>Os05g01430<br>Os05g09640<br>Os05g31610<br>Os06g04810<br>Os06g08154<br>Os06g08690<br>Os06g16070<br>Os06g04370<br>Os07g07230<br>Os07g42626<br>Os08g40090<br>Os08g14940<br>Os08g23290<br>Os08g14990<br>Os08g38560<br>Os09g16000<br>Os10g03420<br>Os10g02990<br>Os10g18990<br>Os10g22890<br>Os10g33080<br>Os10g33110<br>Os10g06740<br>Os10g13970<br>Os10g33130<br>Os10g36270<br>Os11g07270<br>Os11g09130<br>Os11g37050<br>Os11g41100<br>Os11g46980<br>Os12g12740
MAC domain protein	IPRO_Cytolysis and apoptosis	Os02g27480	
NBS-LRR disease resistance protein	Defense response (GO_0006952);<br>Apoptosis(GO_0006915)	Os02g16250<br>Os06g17930	Os11g12050
Oxophytodienoate reductase	Response to biotic stress (GO_0006950, GO_0009607)		Os06g11240
Phagocytosis protein	Phagocytosis (GO_0006909)		Os11g03530

**Annotation**	**GO/IPRO terminology**	**Induced by chilling but not H_2O_2**	**Induced by chilling and H_2O_2**
Pathogen induced protein 2-4	Response to stress (GO_0006950)	Os02g47650	
NB-ARC domain protein	Defense response (GO_0006952); Apoptosis (GO_0006915)	Os01g15580<br>Os01g33810<br>Os03g20840<br>Os05g16200<br>Os06g16790<br>Os06g22460<br>Os08g31780<br>Os10g21400<br>Os10g41900<br>Os11g30060<br>Os12g28040<br>Os12g25170<br>CB096624	Os01g02250<br>Os01g72680<br>Os01g52304<br>Os02g18000<br>Os02g16330<br>Os02g27540<br>Os02g35210<br>Os06g20050<br>Os06g06390<br>Os06g47800<br>Os07g29820<br>Os08g16460<br>Os08g42710<br>Os08g05800<br>Os08g42700<br>Os08g42930<br>Os08g43000<br>Os08g43050<br>Os09g14010<br>Os09g10054<br>Os09g30220<br>Os09g34160<br>Os10g04110<br>Os10g04180<br>Os10g04674<br>Os10g07400<br>Os10g22300<br>Os10g04120<br>Os10g04060<br>Os11g06210<br>Os11g13510<br>Os11g47780<br>Os11g11990<br>Os11g45790<br>Os12g17410<br>Os12g30080<br>Os12g18374<br>Os12g29290<br>Os12g32590
Oxophytodienoate reductase	Response to biotic stress (GO_0006950, GO_0009607)		Os06g11240
Pathogenesis related protein	Defense response to biotic and abiotic stresses (GO_0006952, GO_0009607)	Os09g32280	Os04g50700<br>Os10g11500<br>Os01g14550
Phenylalanine ammonia lyase	Response to stress (GO_0006950)	Os02g41670	Os02g41630<br>Os02g41650
Viral-response protein	IPRO_TMV response	Os09g27260	Os06g13160
Blight resistance protein	Defense to biotic stress (GO_0006952, GO_0009607)		AK069827

Annotation	GO/IPRO terminology	Induced by chilling but not H_2O_2	Induced by chilling and H_2O_2
Senescence-associated protein 5	Defense to biotic stress (GO_0006952, GO_0009607)		Os02g47650<br>AK065366
S-adenosyl-methionine SA methyltransferase	Defense to biotic stress (GO_0006952, GO_0009607)		TA51135_4530
Reticuline oxidase	Response to stress and external stimuli (GO_0006950, GO_0009605)		Os06g35700
SAM dependent carboxyl methyl-transferase	Response to biotic stress (GO_0006950, GO:0009607); IPRO_JA and SA metabolism	Os06g20630	Os01g50480<br>Os06g13560<br>Os06g20790
Sialin	Response to biotic stimulus (GO_0009607)	Os09g38410	
Strictosidine synthase	IPRO_Alkaloid biosynthesis; Response to stress (GO_0006950)		Os01g50330
SOUL heme-binding protein	IPRO_Apoptosis	Os01g63210<br>Os01g11230	Os02g33020
Thaumatin-like protein	Response to biotic stimulus (GO_0009607)		Os10g27280<br>Os11g37940
Wound induced protein WIN2	Defense to fungi and bacteria (GO_0042742, GO_0050832)		Os11g37940
