# Supplementary material for: Transcriptional regulatory network triggered by oxidative signals configures the early response mechanisms of japonica rice to chilling stress
Source: BMC Plant Biol. 2010 Jan 25;10:16. doi: 10.1186/1471-2229-10-16 (PMC2826336; doi:10.1186/1471-2229-10-16)
Supplement: Additional file 10 — Components of the chilling stress transcriptome with possible roles in cellular detoxification and efflux. List of upregulated genes with possible roles in cellular detoxification and efflux classified according to gene ontology. [file 1471-2229-10-16-S10.PDF]

**Additional file 10.** Genes associated with cellular detoxification and efflux processes based on relevant Gene Ontology (GO) and Interpro (IPRO) terminologies.

| Annotation | GO/IPRO terminology | Induced by chilling but not $H_2O_2$ | Induced by chilling and $H_2O_2$ |
|---|---|---|---|
| Transporter protein | Tetracycline:hydrogen antiporter (GO_0015520) | | Os01g16260 |
| Copper amine oxidase | IPRO_Oxidation of xenobiotic amines | Os06g23140<br>Os04g04950<br>Os04g40040 | |
| Diphthamide synthesis protein | IPRO_Resistance to diphtheria toxin | | Os02g57050 |
| Drug transporter protein | Tetracycline:hydrogen antiporter (GO_0015520) | | Os02g57050 |
| Epoxide hydrolase | IPRO_Response to toxin | | CR291891<br>Os10g35490 |
| Exo70 exocyst complex subunit | Exocytosis (GO_0006887) | Os03g33520<br>Os04g58870 | Os08g40840<br>Os01g67820 |
| Exocyst complex component Sec5 | Exocytosis (GO_0006887) | | Os10g27990 |
| Exostosin protein | Exocytosis (GO_0006887) | Os03g01760 | |
| HAD superfamily phosphatase | IPRO_Xenobiotic degradation | Os06g36400 | Os05g10330 |
| HAD-superfamily hydrolase | IPRO_Xenobiotic degradation | Os03g17590 | |
| Haloacid dehalogenase-like hydrolase | IPRO_Xenobiotic degradation | Os10g41930 | Os03g16670<br>Os08g42950 |
| MATE efflux family protein | Drug transporter (GO_0015238) | Os04g40040<br>Os10g13940 | Os08g37432<br>Os08g44870<br>Os10g20350<br>Os10g20470 |
| Metal tolerance protein C3 | IPRO_ Induced by metal toxicity; Cation transporter (GO_0008324) | Os01g62070 | Os01g03914<br>Os01g03914 |
| Multidrug resistance protein 1 | Drug transporter activity (GO_0015238) | Os01g50100 | Os05g47500 |
| Multidrug resistance protein 17 | Drug transporter activity (GO_0015238) | Os01g18670<br>Os05g04610 | |
| Phytochelatin synthase | IPRO_Detoxification; Response to stress (GO_0006950) | Os06g01260<br>Os06g01260 | Os05g34290 |
| Plasma membrane associated protein | Tetracycline:hydrogen antiporter (GO_0015520) | | Os01g50440 |
| Rhodanese | IPRO_Cyanide detoxification | | Os10g39860 |
| Sodium/hydrogen exchanger 3 | Sodium:hydrogen antiporter activity (GO_0015385); Response to abiotic stimulus(GO_0009628) | Os05g05590 | |
| Solute carrier family 2 | Tetracycline:hydrogen antiporter (GO_0015520) | Os05g49260 | |
| Transparent testa-12 protein | Multidrug transporter (GO_0006855) | | Os10g20380 |
| UDP-glucuronic acid UDP-N-acetylgalac tosamine transporter | Tetracycline:hydrogen antiporter (GO_0015520); Response to stress (GO_0006950) | Os06g08860 | |
