# Supplementary material for: Transcriptional regulatory network triggered by oxidative signals configures the early response mechanisms of japonica rice to chilling stress
Source: BMC Plant Biol. 2010 Jan 25;10:16. doi: 10.1186/1471-2229-10-16 (PMC2826336; doi:10.1186/1471-2229-10-16)
Supplement: Additional file 12 — Association between chilling-induced transcription factors and stress-associated QTL. Genomic location of chilling upregulated transcription factors relative to the boundaries of known QTL of rice associated with seedling vigor and stress response. [file 1471-2229-10-16-S12.PDF]

Additional file 12. Location of transcription factors within QTL associated with seedling vigor and stress response.

Trait	QTL ID	Locus ID	Genome Coordinates (bp)	Transcription Factor
COLDTL-1	AQDU006/CQP1	Os01g67480	39538156-39543486	bHLH (bHLH109)
		Os01g64000	37501439-37502958	bZIP (ABRE factor)
		Os01g60020	35021120-35019966	NAC (*NAC68*)
		Os01g58420	34093922-34094629	ERF (*AtERF4*)
		Os01g56690	33041606-33043265	bHLH
		Os01g57580	33599243-33601156	bHLH (bHLH68)
	AQAV003	Os01g09760	5026872-5033629	MYB
		Os01g09080	4563224-4565179	WRKY (*WRKY61*)
OSADJCAP-1	AQDX002	Os01g11910	6459511-6461839	bHLH (bHLH37)
		Os01g09080	4563224-4565179	WRKY (*WRKY61*)
SDLVIG-1	AQFR014/AQFR044	Os01g67480	39538156-39543486	bHLH (bHLH109)
		Os01g64000	37501439-37502958	bZIP (ABRE factor)
		Os01g60020	35021120-35019966	NAC (*NAC68*)
		Os01g58420	34093922-34094629	ERF (*AtERF4*)
	AQFR003	Os01g09760	5026872-5033629	MYB
SLTSN-1	AQEM001/AQEM009	Os01g64000	37501439-37502958	bZIP (*AREB*)
	AQEM008/qRNTQ-1	Os01g60020	35021120-35019966	NAC (*NAC68*)
CHLCN -2	AQCW012	Os02g52780	32270722-32275784	bZIP (*ABI5*)
	AQDB003	Os02g45450	27647111-27647785	ERF (*DREB1A*/*CBF3*)
	AQCW014	Os02g43790	26417556-26416645	ERF (*AtERF13*)
OSADJCAP-2	CQH6, oa2.1	Os02g45450	27647111-27647785	ERF (*DREB1A*/*CBF3*)
		Os02g43790	26417556-26416645	ERF (*AtERF13*)
SDLVIG-2	AQFR004	Os02g45450	27647111-27647785	ERF (*DREB1A*/*CBF3*)
		Os02g43790	26417556-26416645	ERF (*AtERF13*)
	AQFR010	Os02g52780	32275495-32271330	bZIP (*ABI5*)
	AQFR032	Os02g41510	24873941-24873047	Myb (*OsMyb4*)
		Os02g38130	23060334-23057308	NAC (*NAC044*)
CHLCN -3	CQE15	Os03g09170	4760577-4759684	ERF (*AtERF058*)
		Os03g08500	4352514-4351525	ERF (*ERF2*)
	CQE21	Os03g51580	29467207-29469656	bHLH (bHLH118)
	AQCW008	Os03g42100	23387610-23389006	bHLH (bHLH131)
LFRL-3	DQE18	Os03g21030	11918127-1916817	NAC (*GRAB2*)
		Os03g21060	11955043-11953254	NAC
OSADJCAP-3	AQFT003/oa3.11	Os03g21030	11918127-1916817	NAC (*GRAB2*)
		Os03g21060	11955043-11953254	NAC
	CQH12	Os03g15660	8616097-8615321	ERF (*RAP2.1/2.9/2.10*)
SDLVIG-3	AQEP013	Os03g51580	29467207-29469656	bHLH (bHLH18)
CHLCN -4	CQE32	Os04g43680	25625690-25624283	Myb (*OsMyb4*)
	AQHF012/QDg4b	Os04g52090	30720217-30720885	AP2/ERF (*AtERF4*)
COLDTL-4	AQDU001	Os04g52090	30720217-30720885	AP2/ERF (*AtERF4*)
GERMSP-4	AQFE110/grm4.1	Os04g43680	25625690-25624283	Myb (*OsMyb4*)
SDLVIG-4	AQFR036	Os04g52090	30720217-30720885	AP2/ERF (*AtERF4*)
RELWCN-5	DQE50	Os05g37730	21999887-21998835	Myb (*DIVARICATA*)
SDLVIG-5	AQEP005	Os05g46610	26904355-26905534	Myb (*Myb86*)
	AQEP016	Os05g37730	21999887-21998835	Myb (*DIVARICATA*)

Trait	QTL ID	Locus ID	Genome Coordinates	Transcription Factor
SALTSN-6	AQEM002/qSDS-6	Os06g41100	24567291-24560518	bZIP (*TGA10*)
CHLCN-8	CQE69/CQE62	Os08g41320	25970024-25972528	bHLH (bHLH91)
		Os08g38080	23989017-23991063	bHLH (bHLH87)
		Os08g38020	23951753-23951061	bZIP (*HY5*)
		Os08g36740	23061693-23062433	bHLH (bHLH121)
OSADJCAP-8	AQDX013	Os08g38080	23989017-23991063	bHLH (bHLH87)
		Os08g38020	23951753-23951061	bZIP (*AtbZIP48*)
		Os08g36740	23061693-23062433	bHLH (bHLH121)
SDLVIG-8	AQFR050/AQFV024/	Os08g43210	27192436-27191681	ERF (*DREB1B/CBF1*)
	AQFV026/AQFV028/	Os08g43090	27104533-27107970	bZIP (*RF2b*-like)
	AQFFR013	Os08g43070	27090325-27091116	AtMyc2 (bHLH11)
		Os08g42470	26708223-26710453	bHLH (*GBOF-1*)
	AQFV020/AQFV022	Os08g38020	23951753-23951061	bZIP (*AtbZIP48*)
		Os08g36740	23061693-23062433	bHLH (bHLH121)
		Os08g38080	23989017-23991063	bHLH (bHLH87)
CHLCN -9	AQHF039/QDg9	Os09g28440	17305162-17305986	ERF (*B-3 group*)
	CQE76	Os09g36730	21190162-21189250	Myb (*Hv1*)
		Os09g32510	19405779-19, 07978	bHLH
COLDTL-9	AQAV002	Os09g28440	17305162-17305986	ERF (*B-3 group*)
SDLVIG-9	AQEP006	Os09g35020	20399717-20398977	ERF (*DREB1D/CBF4*)
		Os09g35010	20395679-20395023	ERF (*DREB1B/CBF1*)
	AQEP006	Os09g32510	19405779-19407978	bHLH
	AQFR051	Os09g28440	17305162-17305986	ERF (*B-3 group*)
LFRL-9	DQE30/DQE32	Os09g35020	20399717-20398977	ERF (*DREB1D/CBF3*)
		Os09g35010	20395679-20395023	ERF (*DREB1B/CBF1*)
SALTSN-9	AQEM007/qRNC-9	Os09g28440	17305162-17305986	ERF (*B-3 group*)
COLDTL-10	CQAA11	Os10g27360	14105606-14107132	NAC
		Os10g23050	11684489-11686357	bHLH (bHLH45)
GERMSP-11	CQY5/qLTG11	Os11g45850	27233334-27234376	WRKY (*OsWRKY61*)

*COLDTL (cold tolerance), CHLCN (chlorophyll content), GERMSP (speed of germination), LFRL (leaf rolling), OSADJCAP (osmotic adjustment capacity), RELWCN (relative water content), SALTSN (salt sensitivity), SDLVIG (seedling vigor).
